# Introducing a machine learning algorithm for delirium prediction—the Supporting SURgery with GEriatric Co-Management and AI project (SURGE-Ahead)

**DOI:** 10.1093/ageing/afae101

**Published:** 2024-05-22

**Authors:** Samuel Benovic, Anna H Ajlani, Christoph Leinert, Marina Fotteler, Dennis Wolf, Florian Steger, Hans Kestler, Dhayana Dallmeier, Michael Denkinger, Gerhard W Eschweiler, Christine Thomas, Thomas D Kocar

**Affiliations:** Institute of Geriatric Research, Ulm University Medical Center, Ulm, Germany; Agaplesion Bethesda Clinic Ulm, Ulm, Germany; Institute of the History, Philosophy and Ethics of Medicine, Ulm University, Ulm, Germany; Department of Sociology with a Focus on Innovation and Digitalization, Institute of Sociology, Johannes Kepler University Linz, Linz, Austria; Institute of Geriatric Research, Ulm University Medical Center, Ulm, Germany; Agaplesion Bethesda Clinic Ulm, Ulm, Germany; Agaplesion Bethesda Clinic Ulm, Ulm, Germany; DigiHealth Institute, Neu-Ulm University of Applied Sciences, Neu-Ulm, Germany; Institute of Medical Systems Biology, Ulm University, Ulm, Germany; Institute of the History, Philosophy and Ethics of Medicine, Ulm University, Ulm, Germany; Institute of Medical Systems Biology, Ulm University, Ulm, Germany; Institute of Geriatric Research, Ulm University Medical Center, Ulm, Germany; Department of Epidemiology, Boston University School of Public Health, Boston, USA; Institute of Geriatric Research, Ulm University Medical Center, Ulm, Germany; Agaplesion Bethesda Clinic Ulm, Ulm, Germany; Geriatric Center, University Hospital Tübingen, Tubingen, Germany; Department of Psychiatry and Psychotherapy, Tübingen University Hospital, Tübingen, Germany; Department of Psychiatry and Psychotherapy, Tübingen University Hospital, Tübingen, Germany; Department of Geriatric Psychiatry and Psychotherapy, Klinikum Stuttgart, Stuttgart, Germany; Institute of Geriatric Research, Ulm University Medical Center, Ulm, Germany; Agaplesion Bethesda Clinic Ulm, Ulm, Germany

**Keywords:** delirium prediction, machine learning, support vector machine, post-operative delirium, explainable artificial intelligence (AI), older people

## Abstract

**Introduction:**

Post-operative delirium (POD) is a common complication in older patients, with an incidence of 14–56%. To implement preventative procedures, it is necessary to identify patients at risk for POD. In the present study, we aimed to develop a machine learning (ML) model for POD prediction in older patients, in close cooperation with the PAWEL (patient safety, cost-effectiveness and quality of life in elective surgery) project.

**Methods:**

The model was trained on the PAWEL study’s dataset of 878 patients (no intervention, age ≥ 70, 209 with POD). Presence of POD was determined by the Confusion Assessment Method and a chart review. We selected 15 features based on domain knowledge, ethical considerations and a recursive feature elimination. A logistic regression and a linear support vector machine (SVM) were trained, and evaluated using receiver operator characteristics (ROC).

**Results:**

The selected features were American Society of Anesthesiologists score, multimorbidity, cut-to-suture time, estimated glomerular filtration rate, polypharmacy, use of cardio-pulmonary bypass, the Montreal cognitive assessment subscores ‘memory’, ‘orientation’ and ‘verbal fluency’, pre-existing dementia, clinical frailty scale, age, recent falls, post-operative isolation and pre-operative benzodiazepines. The linear SVM performed best, with an ROC area under the curve of 0.82 [95% CI 0.78–0.85] in the training set, 0.81 [95% CI 0.71–0.88] in the test set and 0.76 [95% CI 0.71–0.79] in a cross-centre validation.

**Conclusion:**

We present a clinically useful and explainable ML model for POD prediction. The model will be deployed in the Supporting SURgery with GEriatric Co-Management and AI project.

## Key Points

- POD is a common complication in older patients.
- We aimed to develop an ML model for POD prediction in older patients.
- We developed a linear SVM, capable of accurately predicting POD in older patients.

## Introduction

Delirium is a common complication in older hospitalised patients, with post-operative delirium (POD) occurring in 14–56% of cases [1, 2]. It is associated with increased length of hospital stay, cognitive decline, loss of functional independence, mortality and health care costs [3]. Risk factors and possible causes for the development of POD include age, frailty, multimorbidity and polypharmacy, the duration and type of surgery, history of falls, sensory deprivation, malnutrition and anaemia [4, 5]. Non-pharmacological interventions can significantly decrease the rate of POD, as demonstrated by the Hospital Elder Life Program (HELP) [6] and the ‘Patientensicherheit, Wirtschaftlichkeit und Lebensqualität’ (PAWEL; i.e. patient safety, cost-effectiveness and quality of life) project [7], which focused on cognitive, sensory, social and sleep interventions. As part of delirium prevention, several studies have explored the use of machine learning (ML) in the POD prediction, with receiver operator characteristics (ROC) indicating satisfactory performance of the resulting models [8–10]. A gap however exists for work leveraging the strengths of ML, such as using many features, continuous variables and hyperparameter tuning, while still producing an explainable model.

Using a feature-rich dataset of 880 patients undergoing elective surgery, the PAWEL-R(isk) project recently published a logistic regression (LR) model, yielding highly accurate classification of POD (ROC area under the curve (AUC) = 0.80) [2]. The application of delirium prediction tools is desirable in digital healthcare applications targeting older adults. In the SURGE-Ahead project (Supporting SURgery with GEriatric Co-Management and AI [11]), we aim to develop a digital healthcare application with a dashboard-style user-interface, assisting surgical teams in the care of geriatric patients. One cornerstone of this application will be the prediction of POD.

In this context, the aim of this study was the development of an algorithm for the prediction of POD in the settings relevant for the SURGE-Ahead project. The algorithm should, therefore, be aligned to the following framework: (i) robustness across clinical settings, (ii) a high level of automation and (iii) straightforward explainability that provides both a calibrated estimate of the POD probability and the features that contributed most to that estimate.

## Material and methods

### Data

The present study adheres to the TRIPOD statement for reporting predictive modeling studies (Supplementary Table 5). The data analysed in this project were provided by the PAWEL project. We received data for 899 patients (209 with POD, 690 without POD), aged 70 years or older, who underwent elective surgery at one of five centres in the state of Baden-Wuerttemberg, Germany from June 2017 until January 2019. In the dataset, presence or absence of delirium was determined by repeated ICD-10 adapted Confusion Assessment Method (I-CAM) assessments [12] and a chart review. Consistent with the PAWEL study, participants who withdrew from the study before the first post-operative assessment were excluded from the data analysis. In cases where I-CAM assessments were incomplete and no delirium was detected, we discarded the entire sample, as it was unclear whether delirium did develop later in the study or not, leading to a sample size *n* = 878 (missing *n* = 21, 2.3%). [2] For further information on the dataset, including a list of assessments and detailed procedures, we refer to the PAWEL trial study protocol [13].

### Data preprocessing

Only those PAWEL variables also available in the SURGE-Ahead dataset were considered for the analysis. Polypharmacy was evaluated by counting the number of long-term drugs. A co-morbidity score was calculated by multiplying the pre-existing conditions with their respective values in the Charlson Co-morbidity Index [14]. In detail, the considered co-morbidities were myocardial infarction, congestive heart failure, peripheral vascular disease, cerebrovascular disease, dementia, chronic pulmonary disease (1 point each), liver disease (1 if mild, else 3), diabetes mellitus (1 if without complications, else 2) and renal disease (2 points). Glomerular filtration rate (eGFR) was estimating using the Cockcroft–Gault method [15]. The data were randomly split into a training and a test set with a 4:1 ratio. Missing values (n = 88; 0.7%) were replaced by the mean for continuous and the median for discrete and categorical variables, as defined by the training data. Afterwards, continuous and discrete variables were z-transformed, using the training data as reference. Categorical variables were one-hot encoded to binary variables.

### Model selection

The medico-ethical principles of autonomy, beneficence, non-maleficence and social justice [16] served as the conceptual framework, as well as currently proposed guidelines for algorithmic design by the European Commission [17, 18] and the WHO [19] encompassing transparency, explainability and flexibility/robustness as fundamental values. To reach these goals, we decided to limit ourselves to linear ML models namely an LR and a linear support vector machine (SVM), as opposed to non-linear models that may yield better performance but at the cost of transparency and robustness.

### Target variable

POD, the primary endpoint, was assessed by the I-CAM [12] on days 1–7 after surgery and a chart review at the end of the study.

### Feature selection

For feature selection, we focused on automatically generated data that require little to no human intervention. For explainability, we followed an established guide for interpretable ML data [20]. Furthermore, face validity, as perceived by a delirium expert, played an important role in creating the ML model.

To strike a balance between information gain and complexity of data acquisition, we limited the number of features a priori to 15. This high sample-to-feature ratio is unlikely to trigger the Hughes effect despite the pronounced class imbalance [21]. We additionally explored increasing the number of features to 16. For further information see Supplementary Tables 1–3. Candidate features were selected based on previous ML models [2, 8], literature reviews [4, 5, 22, 23] and expert domain knowledge. During the feature selection process, we focused on explainability, face validity and ease/reliability of data acquisition. The candidate features were: age (months), surgery type (cardiac/other), American Society of Anesthesiologists (ASA) score (score) [24], use of cardio-pulmonary bypass (Yes/No), clinical frailty score (score) [25], cut-to-suture time (minutes), preexisting dementia (Yes/No), eGFR (ml/min), Montreal Cognitive Assessment (MoCA) score (score) [26] (represented as three subscores: memory, orientation and verbal fluency), the custom multimorbidity score as described above (score), number of medications (*n*), post-operative isolation (Yes/No), pre-operative use of benzodiazepines (Yes/No) and/or antipsychotics (Yes/No), falls in the last 3 months (Yes/No), alcohol abuse (Yes/No), anaemia represented by haemoglobin concentration (g/dl), pain as reported by the numeric rating scale ( score), post-operative presence of a urinary catheter system (Yes/No), positive history of delirium (Yes/No) and sensory impairment represented by either impaired hearing or vision (Yes/No). From this set of 23 candidate features, 3 were eliminated due to data quality (sensory impairment, urinary catheter system, pain), 2 due to low variance (alcohol abuse, pre-operative use of antipsychotics) and 1 due to collinearity (cardio-vascular surgery type, collinear with cardio-pulmonary by-pass, r = 0.78; cut-off: 0.7; for a full correlation matrix of the included features, see Supplementary Figure 2) [27]. The remaining 17 features underwent a recursive feature elimination [28], training both an LR and an SVM model and selecting from the 5 features with the smallest coefficients for both models one feature according to our general feature selection criteria described above, until the predefined number of 15 features remained. For a detailed description of the feature selection process, see Supplementary Figure 1.

### Model training

Using the scikit-learn library for python [29] version 1.2.2, two ML models were trained on the training set, an LR and a linear SVM, both using the liblinear [30] implementation. Due to the geometric properties of the hinge loss function, a class weight was applied to the SVM, scaling its loss inversely proportional to the frequency of the respective class. No imbalance correction was performed for the LR, as doing so would likely cause performance loss and miscalibration [31]. The L2 regularisation hyperparameter C was chosen from a range of 17 values between 28 and 2–8, evenly spaced on a logarithmic scale, by a leave-one-out cross-validation optimising for accuracy. The ‘max_iter’ hyperparameter was set to 10^9^ to ensure convergence. For the remaining hyperparameters, the default values from the scikit-learn library were used. After training, the SVM was calibrated to the training data using Platt Scaling [32]. The final model was determined by the better ROC AUC in the test set. In the final model, ROC AUC, F1 score, sensitivity and specificity were calculated, as well as their 95% confidence interval, determined by bootstrapping with 1000 iterations [33]. For metrics requiring a binary output, the sign function of the decision function z was used to set the decision boundary. Coefficients of the final model and, in case of the SVM, Platt Scaling parameters are reported. Possible algorithmic bias of the model by sex and native language (there were no data for ethnicity) was investigated using the Aequitas framework for python [34].

## Results

### Study population

For the ML model, we used data from 878 patients with a mean age (SD) of 77.8 (±4.91) collected in the PAWEL-R study. POD occurred in 209 (23.8%) of cases, with 171 (24.4%) of cases in the training set. Pre-existing dementia was present in 14 (1.6%) of patients. The most common ASA classification was III (*n* = 542, 61.7%). For a summary of the population characteristics, see Table 1 and the PAWEL-R study [2], which was our data source.

**Table 1:** **Study population characteristics (*n* = 877).** Mean and standard deviation (SD) are given for continuous and discrete variables and the number of observations (*N*) for binary variables. ASA = American Society of Anesthesiologist, MoCA = Montreal Cognitive Assessment, eGFR = estimated glomerular filtration rate (Cockcroft–Gault formula).

	With Delirium (*N* = 209)		Without Delirium (*N* = 669)		Total
Variable	Mean ± SD	*N* (%)	Mean ± SD	*N* (N%)	Missing (%)
Demographics					
Age, years	78.3 ± 5.21		77.6 ± 4.79		0 (0.0%)
Male		118 (56.5%)		325 (48.6%)	0 (0.0%)
Surgical setting					0 (0.0%)
Cardiovascular		120 (57.4%)		211 (31.5%)	
Orthopaedic		74 (35.4%)		388 (58.0%)	
Abdominal		12 (5.7%)		59 (8.8%)	
General		3 (1.4%)		11 (1.6%)	
ASA	3.2 ± 0.58		2.7 ± 0.59		12 (1.4%)
I		1 (0.5%)		13 (1.9%)	
II		18 (8.6%)		208 (31.1%)	
III		135 (64.6%)		407 (60.8%)	
IV		52 (24.9%)		32 (4.8%)	
Cardio-pulmonary bypass		95 (45.7%)		144 (21.6%)	4 (0.5%)
Clinical Frailty Scale	3.9 ± 1.48		3.5 ± 1.29		9 (1.0%)
Cut-to-suture time, minutes	190.7 ± 100.1		133.0 ± 73.61		1 (0.1%)
Dementia		13 (6.2%)		1 (0.1%)	0 (0.0%)
eGFR, ml/min	66.3 ± 22.99		70.7 ± 22.52		35 (4.0%)
MoCA					
Memory	1.7 ± 1.6		2.4 ± 1.65		7 (0.8%)
Orientation	5.6 ± 0.94		5.9 ± 0.32		7 (0.8%)
Verbal fluency		53 (25.7%)		257 (38.6%)	7 (0.8%)
Multimorbidity, score	1.6 ± 1.53		1.1 ± 1.26		0 (0.0%)
Number of medications	7.1 ± 3.48		5.7 ± 3.27		0 (0.0%)
Post-OP isolation		8 (3.8%)		13 (2.0%)	5 (0.6%)
Pre-OP benzodiazepines		58 (27.8%)		156 (23.3%)	0 (0.0%)
Recent fall		50 (24.0%)		98 (14.6%)	1 (0.1%)

### Features

Our models included the following features: ASA score, multimorbidity, cut-to-suture time, estimated glomerular filtration rate, polypharmacy, use of cardio-pulmonary bypass, the MoCA subscores ‘memory’, ‘orientation’ and ‘verbal fluency’, preexisting dementia, clinical frailty score, age, recent falls, post-operative isolation and pre-operative benzodiazepines.

### Model choice

The LR model gave an ROC AUC of 0.80 in the test set. The linear SVM gave an ROC AUC of 0.81 in the test set. There was very little difference in performance in terms between the two models. The linear SVM was chosen as the final model.

### Model performance

**Figure 1:**
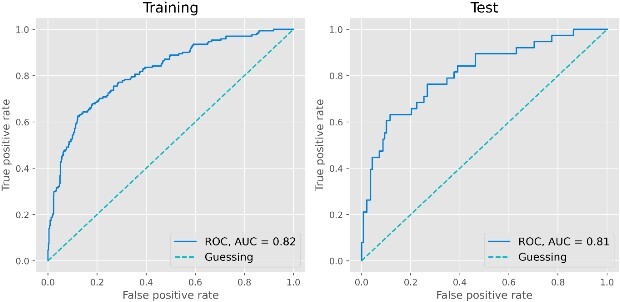
**Receiver operating characteristic curves of the linear SVM delirium prediction algorithm.** Left: training set (*n* = 702), right: test set (*n* = 176). SVM = support vector machine, ROC = receiver operating characteristics, AUC = area under the curve.

The final model gave an ROC AUC of 0.82 [0.78–0.85] in the training set and 0.81 [0.71–0.88] in the test set (see Figure 1). F1 score was 0.58 [0.52–0.64] in the training set and 0.54 [0.41–0.65] in the test set. Sensitivity was 0.71 [0.64–0.77] in the training set and 0.68 [0.52–0.82] in the test set. Specificity was 0.76 [0.73–0.80] in the training set and 0.76 [0.69–0.83] in the test set. For the confusion matrix for the model see Supplementary Table 6.

Dementia was the most predictive for POD, followed by necessity of a cardio-pulmonary bypass during surgery and the cut-to-suture time in the training set. For a list of all 15 features and their respective coefficients, see Table 2 and Figure 1. Note that Table 2 contains all information needed for the SVM decision function z, from which the present delirium prediction ML model can be rebuilt. In a vectorised form, z can be denoted as the dot-product of the column vectors x (preprocessed features) and θ (coefficients):


$$ z(x)={x}^T\theta $$

**Table 2:** **Tabular representation of the SVM decision function z(X).** Continuous and discrete variables are normalised (μ = mean, σ = SD), all categorical variables are binary. The scaled features are multiplied by their respective coefficient θ and then added together to obtain the decision function’s output z. If z is positive, the model predicts a possible delirium (signum function). The features’ units are presented in parentheses, their imputed default value (used to handle missing data) in square bracket. ASA = American Society of Anesthesiologists, eGFR = estimated glomerular filtration rate, MoCA = Montreal Cognitive Assessment.

Z =	Σ	θ_x_	× (	x			−		μ_x_	) ÷	σ_x_
Z =		2.53	× (	Dementia (Y/N)	[	0	]			)	
	+	0.53	× (	Cardio-Pulmonary Bypass (Y/N)	[	0	]			)	
	+	0.53	× (	Cut-to-Suture Time (minutes)	[	146	]	−	146.05	) ÷	85.29
	+	0.42	× (	Pre-OP Benzodiazepines (Y/N)	[	0	]			)	
	+	0.39	× (	ASA Class (score)	[	3	]	−	2.81	) ÷	0.6
	+	0.31	× (	Recent Fall (Y/N)	[	0	]			)	
	+	0.21	× (	Clinical Frailty Scale (score)	[	3	]	−	3.62	) ÷	1.37
	+	0.15	× (	Post-OP Isolation (Y/N)	[	0	]			)	
	+	0.14	× (	Multimorbidity (score)	[	1	]	−	1.27	) ÷	1.35
	+	0.11	× (	Number of Medications (n)	[	6	]	−	6.09	) ÷	3.42
	+	0.10	× (	Age (months)	[	934	]	−	934.47	) ÷	58.86
	−	0.03	× (	eGFR (Cockcroft–Gault) (ml/min)	[	69	]	−	69.21	) ÷	22.39
	−	0.26	× (	MoCA Verbal Fluency (score)	[	0	]			)	
	−	0.27	× (	MoCA Orientation (score)	[	6	]	−	5.85	) ÷	0.56
	−	0.34	× (	MoCA Memory (score)	[	2	]	−	2.24	) ÷	1.66
	−	0.61		(Intercept)							

Platt Scaling maps the decision function z to a corresponding probability (P) for delirium (y = 1), by the following formula:


$$ P\left(y=1|z\right)=\frac{1}{1+{e}^{-0.97z+1.07}} $$

The bias analysis showed no significant bias for sex and native language. For detailed results, see Supplementary Table 4.

## Discussion

### Delirium prediction

We present an ML model for the prediction of POD from pre-operative data composed of 15 features, which are commonly routinely assessed during admission. The model yields good performance in the test data (ROC AUC 0.81) and slightly outperforms the PAWEL-R model trained on the same data. During feature selection and model training, we emphasised the importance of robustness, a high level of automation, straightforward explainability and ethical principles. In turn, we focused on features that are either already routinely assessed in a clinical setting or associated with only minor costs, both in time and resources. The present work is embedded in the SURGE-Ahead project [11]. As opposed to the PAWEL-R model that focused on binary input variables to enable manual calculation, the present model can be expressed as a multivariate regression with a calibrated probabilistic output (see Table 2). This not only allows the implementation in any digital application as a basic mathematical function, but also provides an automated approach towards POD risk estimation if all data can be made routinely available.

The identified coefficients generally align with existing studies, placing importance on pre-existing dementia, ASA class, surgery type and complexity and cognitive impairment (quantified using the MoCA assessment in our case). Cut-to-suture time was also assigned a large coefficient in our model. Sadly, we could not investigate several known predictors for POD, such a pre-existing psychiatric conditions, as those features were not available in our dataset.

### Medico-ethical principles

In what follows, we briefly outline the commonly applied medico-ethical principles by Beauchamp and Childress and their transfer into ethical guidelines focused on artificial intelligence issued by the WHO and European Commission [18, 19]. With regards to ML, transparency and explainability are substantial values [35]. The intended purposes of those values, avoiding harm and facilitating informed decision making, intersect with the medico-ethical principles of non-maleficence and respect for autonomy, respectively [36]. In addition, easy-to-use prediction models can facilitate the targeted use of delirium prevention interventions among those with a high risk for delirium in a resource-limited health care system, consistent with the medico-ethical principle of justice [37]. In patients with mild cognitive impairment, assessment of the three MoCA-subscores has advantages beyond POD prediction, such as serving as a solid foundation to determine consent capacity [38]. Furthermore, the model has been screened for potential sex and native language bias and does not disadvantage patients based on their sex or native language. In regards to age (>70), the demographic of the training data is representative of the target population of the SURGE-Ahead project [11].

**Figure 2:**
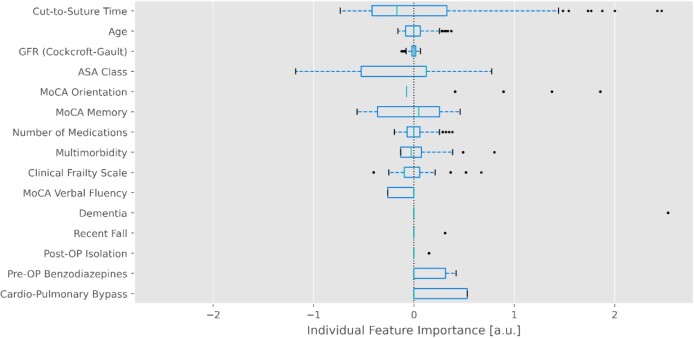
**Distribution of the individual feature importance in the training set. In the linear** SVM**,** the individual feature importance is determined by the element-wise multiplication of the coefficient and feature vectors. As the distribution of the individual feature importance is approximately centred around 0, it is possible to represent the individual feature importance in numerical terms, where positive numbers indicate a higher risk of delirium and negative numbers a lower risk (see *x*-axis).

Both LR and the linear SVM are transparent and highly explainable ML models, which enabled us to display features that were important for a particular prediction. In linear models, the element-wise product of the feature vector and the parameter vector gives the contribution from each input feature to the model’s prediction. The product of any feature and its respective coefficient is a scalar with a certain magnitude and sign. A positive scalar shifts the prediction towards the positive class (POD) and a negative scalar towards the negative class (no POD). In Figure 2, we present a boxplot of the distribution of these feature importance scalars in the test set, providing insights into how certain features have influenced the outcome of individual predictions. With a large interquartile range, the cut-to-suture-time, ASA score and MoCA orientation score generally contribute most to the individual predictions. As our input features are centred around 0, which can serve as a reference frame, presenting numerical values as a proxy for individual feature importance is possible. Deriving individual feature importance from the element-wise product of the feature vector and the parameter vector simplifies explainablity when implemented in a digital application, where, as an example, the top contributors to the prediction could be highlighted for the user.

To sustain awareness that experience-based clinical knowledge is essential to navigate the ‘complex and inherently indeterminable phenomena’ that occur in human interaction [39], we decided to relate the estimated risk in the format of a percentage via Platt Scaling. In terms of respecting patients’ autonomy and sustaining participative treatment planning, face validity and explainability of predictions promote transparent and understandable communication. Potential asymmetries of precision in predictions for different patient subgroups as well as user acceptance and feedback from both physicians and patients will remain important areas of close observation throughout deployment [40].

### Limitations

First, using the PAWEL-R study as a source for both the data as well as identifying features comes with a risk for information spillover, biassing feature selection towards variables identified in that study. To investigate this point, we conducted an additional internal-external cross-validation at the centre level, fitting the model to all but one centres and testing the model on the held-out centre [41]. In this analysis, the weighted average ROC AUC was 0.82 [95% CI 0.80–0.84] in the training set and 0.76 [95% CI 0.71–0.79] in the test set (see Supplementary Figure 3). Second, it is common for performance metrics of clinical prediction tools to drop significantly when tested on external data [10, 42]. In regard to this, an external validation is planned with data from an observational study that recently started within the SURGE-Ahead project and is expected to end in early 2024 [11]. Third, our model was calibrated to the entire sample, however, prevalence of delirium differs in various settings. Prospectively, separate calibrations for different settings could be considered. Additionally, the PAWEL cohort included only data from Germany, possibly causing racial bias, as well as only elective surgical procedures, possibly having excluded individuals accounted too frail for elective surgery. Fourth, some of the features and assessments used by the model might be too time-intensive for becoming routine clinical practice. This mainly affects the three MoCA subscores, which would require the entire MoCA to be conducted. A comprehensive way to assess these subscores was proposed by Wong and colleagues [43] in a modified version of the MoCA, which requires ~5 min. With the exception of the falls anamnesis, clinical frailty scale and the MoCA 5-min assessment [43], all features are routine data that could be extracted from the patient’s electronic medical record using an automated pipeline, the development of which is part of the SURGE-Ahead project [11]. In addition, the proposed POD prediction algorithm is capable of handling missing data by simple using the median/mean of the training data (see Table 2), albeit at the cost of accuracy. Fifth, for pre-operative delirium prediction, the cut-to-suture time is *per se* not available. At this timepoint the planned cut-to-suture time can be estimated either by the institution- and/or procedure-specific average, or by the surgeon pre-operatively. Additionally, the delirium prediction can be updated immediately after the operation, using the real cut-to-suture time. Sixth, it is plausible that we could have achieved better performance metrics by having more samples or using non-linear models. Using a far larger dataset, Bishara and colleagues [44] were able to create two linear models with ROC AUC scores comparable to ours, but also two non-linear models—a neural network and XGBoost—reaching ROC AUC scores of 0.84 and 0.85, respectively. In the present study, we favoured the robustness, transparency and explainability of linear models over the slightly better performance of non-linear models.

## Conclusion

In summary, we developed an internally validated linear SVM ML model, capable of accurately predicting POD in older patients. If used as a screening tool in a clinical setting, the presented POD prediction algorithm could increase the efficiency and overall feasibility of POD prevention programs.

## Supplementary Material

aa-23-1838-File003_afae101

aa-23-1838-File002_afae101
