# Supplementary material for: Introducing a machine learning algorithm for delirium prediction—the Supporting SURgery with GEriatric Co-Management and AI project (SURGE-Ahead)
Source: Age Ageing. 2024 May 22;53(5):afae101. doi: 10.1093/ageing/afae101 (PMC11110913; doi:10.1093/ageing/afae101)
Supplement: aa-23-1838-File003_afae101 [file aa-23-1838-file003_afae101.docx]

Introducing a machine learning algorithm for Delirium prediction – the Supporting SURgery with GEriatric Co-Management and AI project (SURGE-Ahead)

Supplementary Material

Table of Contents

[Exploratory Analysis 2](#_Toc165302715)

[Methods 2](#_Toc165302716)

[Results 2](#_Toc165302717)

[Discussion 2](#_Toc165302718)

[Supplementary Figures and Tables 3](#_Toc165302719)

[Supplementary Tables 3](#_Toc165302720)

[Supplementary Table 1: List of Features used in the Exploratory Analysis 3](#_Toc165302721)

[Supplementary Table 2: Engineered Features 4](#_Toc165302722)

[Supplementary Table 3: Results from the exploratory analysis 5](#_Toc165302723)

[Supplementary Table 4: Bias analysis 20](#_Toc165302724)

[Supplementary Table 5: TRIPOD Statement 21](#_Toc165302725)

[Supplementary Table 6: Confusion matrix 23](#_Toc165302726)

[Supplementary Figures 24](#_Toc165302727)

[Supplementary Figure 1: Feature Selection Process displayed as a flow chart 24](#_Toc165302728)

[Supplementary Figure 2: Correlation Matrix of the included features 26](#_Toc165302729)

[Supplementary Figure 3: Internal cross-validation based on location 27](#_Toc165302730)

# Exploratory Analysis

## Methods

As an additional exploratory analysis, we searched for a potential 16th feature by iteratively adding a single feature to our feature set, as well as trying additional engineered features. We tested all features available to the Surge-AHEAD project, without considering explainability or plausibility (see **Supplementary Table 3** for a complete list of all features tested). The models were trained on 75% (n=526) of the training set, with the remaining 25% (n=176) acting as a test set. Performance of the resulting models was evaluated by measuring the ROC AUC in both sets. The process was performed with both the LR and the SVM. This exploratory analysis had no influence on the final model.

## Results

None of the additional features lead to a significant improvement of the model, as demonstrated by the overlapping 95% confidence intervals. The SVM trained on the subset of the training data had a performance (ROC AUC [95%CI]) of 0.84 [0.79 – 0.87] (training set) and 0.75 [0.66 – 0.83] (test set). The added feature with the best performance was the postoperative use of a urinary catheter with performance metrics (ROC AUC [95%CI]) of 0.85 [0.80 – 0.88] (training set), 0.77 [0.68 – 0.84] (test set). For the results of all feature candidates, as well as for the results of the LR, see **Supplementary Table 3**.

## Discussion

In the exploratory analysis, the only feature showing promise in further improving the POD prediction was the post-operative presence of a urinary catheter system (see **Supplementary Table 3**). However, our feature selection process excluded this feature because the presence of a urinary catheter system was not known preoperatively from the given data. The postoperative presence of a urinary catheter system might also be an indicator of a long and severe surgery, multimorbidity, extreme frailty, decubiti or cachexia. Future studies could further investigate the relationships between urinary catheter systems and the development of POD with the focus on pre-operatively available information.

# Supplementary Figures and Tables

## Supplementary Tables

Supplementary Table 1: List of Features used in the Exploratory Analysis**.** The following features were available to the SURGE-Ahead and were therefore considered in the Exploratory Analysis. Features not taken directly from the PAWEL dataset are described in **Supplementary Table 2**.

| **Feature category** | **List of features in category** |
| --- | --- |
| Basic patient data | "sex", "weight", "height" |
| Anamnesis | “pain", "married", "separated from spouse", "unmarried", "divorced", "widowed", "has children", "number of children", "living alone", "living with children", "living with spouse", "living with other family members", "living in a retirement home", "living in a nursing home", "living with relatives", "nicotine abuse", "alcohol abuse", "body mass index", "lost more than 3 kg", "lost 1-3 kg", "lost less than 1 kg", "amount of recent falls", "hearing aid", "seeing aid", "bad memory", "has Pflegegrad", "level of Pflegegrad", "Pflegegrad is requested", "source of anamnesis: patient", "source of anamnesis: chart", "source of anamnesis: relatives", "source of anamnesis: other" |
| Prior conditions | "myocardial infarction", "congestive heart failure", "chronical lung disease", "gastroduodenal ulcer", "renal disease", "diabetes without complications", "diabetes with complications", "diabetes, any kind", "cancer", "unconciousness", "seizure", "parkinson's disease", "hypertension", "hypotension", "arhrosis of the hand", "other arthrosis", "stroke", "hard of hearing", "mild liver disease", "other liver disease", "liver disease, any kind", "cerebrovascular disease", "peripheral vascular disease", "vascular disease", "neurological condition", "disturbance of conciousness", "cardiovascular condition" |
| Clinical chemistry | "haemoglobin", "sodium", "creatinine", "C-reactive protein" |
| Anesthesiological data | "regional anesthesia", "general anesthesia using gas", "general anesthesia using IVs", "anesthesia with intubation", "anesthesia with larinx mask", "spinal anesthesia", "pre-operative opiods", "time under genral anesthesia" |
| Surgical data | "surgery setting: cardiovascular", "surgery setting: orthopedic", "surgery setting: visceral", "surgery setting: general", "surgery type: joint", "surgery type: spine", "surgery type: vascular", "surgery type: heart", "surgery type: lungs", "surgery type: abdominal", "surgery type: urological", "surgery type: other" |
| Assesments and Scores | "MoCA", "digitspan backwards test", "PHQ-4 nervous", "PHQ-4 worrying", "PHQ-4 little interest", "PHQ-4 feeling down", "PHQ-4", "PHQ-4 depression", "PHQ-4 anxiety", "EQ-5D-5L mobility", "EQ-5D-5L self-care", "EQ-5D-5L usual activities", "EQ-5D-5L pain", "EQ-5D-5L anxiety", "EQ-5D-5L visual analog scale", "EQ-5D-5L", "SF-12 general health", "SF-12 pain", "SF-12 social contacts", "SF-12", "Barthel feeding", "Barthel bathing", "Barthel grooming", "Barthel dressing", "Barthel bowel control", "Barthel bladder control", "Barthel toilet use", "Barthel transfers", "Barthel mobility", "Barthel stairs", "Barthel", "mobile at home", "mobile outside", "CHA2DS2-VASc score" |

Supplementary Table 2: Engineered Features**.** Features not directly present in the PAWEL datatset were constructed from those that were. The table doesn’t list engineered features already described in the main manuscript.

| **Feature** | **Type** | **Description** |
| --- | --- | --- |
| "alcohol abuse" | binary | consumption of alcoholic beverages more than 5 times per week |
| "living with relatives” | binary | any of "living with spouse", "living with children", "living with other family members" |
| neurological condition" | binary | any of "dementia", "seizure", "parkinson's disease", "stroke", "positive history of delirium", "cerebrovascular disease" |
| "disturbance of conciosness" | binary | any of "unconciousness", "seizure", "positive history of delirium" |
| "cardiovascular condition" | binary | any of "myocardial infarction", "heart failure", "hypertension", "hypotension", "diabetes", "vascular disease", "cerebrovascular disease", "peripheral vascular disease" |
| "sensory deprivarion" | binary | any of "hearing aid", "seeing aid", "hard of hearing" |
| "time under general anesthesia" | continuous | "cut to suture time" if general anesthesia was performed, else 0 |
| "PHQ-4" | ordinal | sum of "PHQ-4 nervous", "PHQ-4 worrying", "PHQ-4 little interest", "PHQ-4 feeling down |
| "PHQ-4 anxiety" | binary | 1 if the sum of "PHQ-4 nervous", "PHQ-4 worrying" is greater than 2, else 0 |
| "PHQ-4 depression" | binary | 1 if the sum of "PHQ-4 little interest", "PHQ-4 feeling down", else 0 |
| "Barthel" | ordinal | sum of "Barthel feeding", Barthel bathing", "Barthel grooming", "Barthel dressing", "Barthel bowel control", "Barthel bladder control", "Barthel toilet use", "Barthel transfers", "Barthel mobility", "Barthel stairs" |
| "SF-12" | ordinal | sum of "SF-12 general health", "SF-12 pain", "SF-12 social contacts" |
| "EQ-5D-5L" | continuous | composite score using the "German Value Set" (https://link.springer.com/article/10.1007/s40273-018-0615-8) |
| "MoCA" | ordinal | sum of "MoCA memory", "MoCA verbal fluency", "MoCA orientation" |

Supplementary Table 3: Results from the exploratory analysis**.** We searched for a potential 16th feature by iteratively adding a single feature from the entire dataset to the feature set, as well as trying additional engineered features. We tested all features available to the Surge-AHEAD project, without considering explainability or plausibility. The results were compared to a model trained on 75% of the training set using the 15 features identified during feature selection; the results of this base model is given in **bold**. The remaining 25% of the training set acted as the test set for this analysis. The 5 features which gave the highest ROC-AUC for the logistic regression are given in bold as well.

|  | **Linear support vector machine** | | | | | | **Logistic regression** | | | | | |
| --- | --- | --- | --- | --- | --- | --- | --- | --- | --- | --- | --- | --- |
|  | **Training set** | | | **Test set** | | | **Training set** | | | **Test set** | | |
| **Feature** | **ROC-AUC** | **Sensitivity** | **Specifity** | **ROC-AUC** | **Sensitivity** | **Specifity** | **ROC-AUC** | **Sensitivity** | **Specifity** | **ROC-AUC** | **Sensitivity** | **Specifity** |
| Postoperative urinary catheter | 0.85  [0.80 – 0.88] | 0.76  [0.68 – 0.83] | 0.73  [0.69 – 0.77] | 0.77  [0.68 – 0.84] | 0.65  [0.51 – 0.78] | 0.72  [0.63 – 0.79] | 0.83  [0.78 – 0.87] | 0.42  [0.33 – 0.50] | 0.95  0.92 – 0.97] | 0.77  [0.68 – 0.84] | 0.35  [0.22 – 0.49] | 0.93  [0.87 – 0.97] |
| Cardiovascular disease | 0.84  [0.79 – 0.88] | 0.75  [0.67 – 0.82] | 0.77  [0.72 – 0.81] | 0.77  [0.68 – 0.84] | 0.63  [0.49 – 0.76] | 0.77  [0.69 – 0.84] | **0.81**  **[0.77 – 0.85]** | **0.33**  **[0.25 – 0.42]** | **0.97**  **[0.95 – 0.98]** | **0.79**  **[0.70 – 0.86]** | **0.26**  **[0.15 – 0.40]** | **0.98**  **[0.94 – 0.99]** |
| Sodium | 0.84  [0.79 – 0.88] | 0.74  [0.65 – 0.81] | 0.75  [0.71 – 0.79] | 0.77  [0.68 – 0.84] | 0.63  [0.49 – 0.76] | 0.78  [0.70 – 0.84] | **0.83**  **[0.78 – 0.86]** | **0.43**  **[0.35 – 0.52]** | **0.95**  **[0.92 – 0.97]** | **0.78**  **[0.69 – 0.85]** | **0.35**  **[0.22 – 0.50]** | **0.94**  **[0.88 – 0.97]** |
| Surgery type: vascular | 0.84  [0.79 – 0.88] | 0.74  [0.65 – 0.81] | 0.78  [0.74 – 0.82] | 0.76  [0.67 – 0.84] | 0.61  [0.46 – 0.75] | 0.75  [0.67 – 0.82] | 0.83  [0.78 – 0.87] | 0.42  [0.33 – 0.50] | 0.95  [0.93 – 0.97] | 0.77  [0.68 – 0.85] | 0.35  [0.22 – 0.49] | 0.93  [0.87 – 0.97] |
| Hard of hearing | 0.84  [0.79 – 0.87] | 0.76  [0.68 – 0.83] | 0.74  [0.70 – 0.78] | 0.76  [0.67 – 0.84] | 0.67  [0.53 – 0.80] | 0.75  [0.67 – 0.82] | 0.83  [0.78 – 0.87] | 0.42  [0.34 – 0.51] | 0.96  [0.93 – 0.97] | 0.77  [0.68 – 0.84] | 0.35  [0.22 – 0.49] | 0.93  [0.87 – 0.97] |
| Barthel transfers | 0.83  [0.79 – 0.87] | 0.74  [0.66 – 0.82] | 0.76  [0.72 – 0.80] | 0.76  [0.67 – 0.84] | 0.63  [0.49 – 0.76] | 0.75  [0.66 – 0.82] | 0.83  [0.78 – 0.86] | 0.42  [0.34 – 0.51] | 0.95  [0.92 – 0.97] | 0.77  [0.68 – 0.84] | 0.35  [0.22 – 0.49] | 0.93  [0.87 – 0.97] |
| Chronical lung disease | 0.83  [0.79 – 0.87] | 0.75  [0.67 – 0.82] | 0.75  [0.71 – 0.79] | 0.76  [0.67 – 0.84] | 0.65  [0.51 – 0.78] | 0.75  [0.67 – 0.82] | 0.83  [0.78 – 0.87] | 0.42  [0.33 – 0.50] | 0.95  [0.92 – 0.97] | 0.77  [0.68 – 0.84] | 0.35  [0.22 – 0.49] | 0.93  [0.87 – 0.97] |
| Unconciousness | 0.84  [0.80 – 0.88] | 0.77  [0.69 – 0.84] | 0.78  [0.74 – 0.82] | 0.76  [0.67 – 0.84] | 0.63  [0.48 – 0.77] | 0.79  [0.71 – 0.86] | **0.84**  **[0.79 – 0.88]** | **0.45**  **[0.36 – 0.54]** | **0.95**  **[0.92 – 0.97]** | **0.77**  **[0.68 – 0.85]** | **0.37**  **[0.24 – 0.51]** | **0.93**  **[0.87 – 0.97]** |
| Hypertension | 0.84  [0.79 – 0.88] | 0.74  [0.66 – 0.81] | 0.77  [0.72 – 0.81] | 0.76  [0.67 – 0.84] | 0.63  [0.49 – 0.76] | 0.76  [0.68 – 0.83] | 0.84  [0.79 – 0.87] | 0.44  [0.35 – 0.53] | 0.95  [0.92 – 0.96] | 0.77  [0.68 – 0.85] | 0.35  [0.22 – 0.49] | 0.92  [0.87 – 0.96] |
| Disturbance of conciosness | 0.84  [0.80 – 0.88] | 0.78  [0.69 – 0.84] | 0.75  [0.71 – 0.79] | 0.76  [0.67 – 0.84] | 0.65  [0.51 – 0.78] | 0.78  [0.70 – 0.84] | 0.84  [0.80 – 0.88] | 0.45  [0.36 – 0.54] | 0.95  [0.92 – 0.97] | 0.77  [0.68 – 0.85] | 0.46  [0.31 – 0.60] | 0.93  [0.87 – 0.97] |
| Peripheral vascular disease | 0.83  [0.79 – 0.87] | 0.72  [0.63 – 0.79] | 0.75  [0.70 – 0.79] | 0.76  [0.67 – 0.84] | 0.61  [0.46 – 0.75] | 0.76  [0.68 – 0.83] | 0.83  [0.79 – 0.87] | 0.43  [0.35 – 0.52] | 0.95  [0.92 – 0.97] | 0.76  [0.67 – 0.84] | 0.35  [0.22 – 0.49] | 0.93  [0.87 – 0.97] |
| Gastroduodenal ulces | 0.83  [0.79 – 0.87] | 0.77  [0.69 – 0.84] | 0.73  [0.69 – 0.77] | 0.76  [0.67 – 0.84] | 0.65  [0.51 – 0.79] | 0.73  [0.65 – 0.80] | 0.84  [0.79 – 0.87] | 0.41  [0.33 – 0.50] | 0.95  [0.93 – 0.97] | 0.77  [0.68 – 0.84] | 0.37  [0.24 – 0.51] | 0.93  [0.88 – 0.97] |
| Surgery type: joint | 0.84  [0.79 – 0.87] | 0.76  [0.68 – 0.83] | 0.77  [0.73 – 0.81] | 0.76  [0.67 – 0.84] | 0.63  [0.48 – 0.77] | 0.77  [0.69 – 0.83] | 0.83  [0.78 – 0.86] | 0.42  [0.33 – 0.50] | 0.95  [0.92 – 0.97] | 0.77  [0.67 – 0.84] | 0.35  [0.22 – 0.49] | 0.93  [0.87 – 0.97] |
| Barthel dressing | 0.84  [0.79 – 0.87] | 0.74  [0.66 – 0.81] | 0.79  [0.74 – 0.82] | 0.76  [0.67 – 0.84] | 0.61  [0.46 – 0.75] | 0.77  [0.69 – 0.84] | 0.83  [0.79 – 0.87] | 0.47  [0.39 – 0.56] | 0.95  [0.93 – 0.97] | 0.77  [0.68 – 0.84] | 0.37  [0.24 – 0.51] | 0.92  [0.87 – 0.96] |
| Mild liver disease | 0.84  [0.79 – 0.88] | 0.75  [0.67 – 0.82] | 0.77  [0.72 – 0.81] | 0.76  [0.67 – 0.84] | 0.61  [0.46 – 0.75] | 0.76  [0.68 – 0.83] | 0.83  [0.79 – 0.87] | 0.42  [0.34 – 0.51] | 0.95  [0.92 – 0.96] | 0.77  [0.67 – 0.84] | 0.35  [0.22 – 0.49] | 0.93  [0.87 – 0.97] |
| Seeing aid | 0.84  [0.79 – 0.87] | 0.76  [0.68 – 0.83] | 0.75  [0.71 – 0.79] | 0.76  [0.67 – 0.84] | 0.63  [0.48 – 0.76] | 0.76  [0.68 – 0.83] | **0.82**  **[0.77 – 0.86]** | **0.45**  **[0.36 – 0.54]** | **0.95**  **[0.93 – 0.97]** | **0.78**  **[0.69 – 0.85]** | **0.33**  **[0.20 – 0.47]** | **0.93**  **[0.88 – 0.97]** |
| C-reactive protein | 0.84  [0.79 – 0.88] | 0.74  [0.66 – 0.81] | 0.76  [0.72 – 0.80] | 0.76  [0.67 – 0.84] | 0.63  [0.49 – 0.76] | 0.75  [0.67 – 0.82] | **0.83**  **[0.78 – 0.87]** | **0.42**  **[0.33 – 0.50]** | **0.95**  **[0.93 – 0.97]** | **0.77**  **[0.68 – 0.85]** | **0.35**  **[0.22 – 0.49]** | **0.93**  **[0.87 – 0.97]** |
| Surgery type: spine | 0.84  [0.79 – 0.88] | 0.74  [0.66 – 0.81] | 0.75  [0.70 – 0.79] | 0.76  [0.67 – 0.83] | 0.59  [0.44 – 0.73] | 0.77  [0.69 – 0.84] | 0.84  [0.79 – 0.87] | 0.43  [0.35 – 0.52] | 0.94  [0.91 – 0.96] | 0.76  [0.67 – 0.84] | 0.37  [0.24 – 0.51] | 0.93  [0.87 – 0.97] |
| Surgery type: other | 0.84  [0.80 – 0.88] | 0.76  [0.68 – 0.83] | 0.77  [0.72 – 0.81] | 0.76  [0.67 – 0.83] | 0.61  [0.46 – 0.75] | 0.75  [0.67 – 0.82] | 0.84  [0.79 – 0.87] | 0.43  [0.35 – 0.52] | 0.95  [0.92 – 0.96] | 0.76  [0.67 – 0.84] | 0.35  [0.22 – 0.49] | 0.93  [0.87 – 0.97] |
| Barthel bathing | 0.83  [0.79 – 0.87] | 0.76  [0.68 – 0.83] | 0.76  [0.71 – 0.80] | 0.76  [0.67 – 0.84] | 0.61  [0.46 – 0.75] | 0.73  [0.65 – 0.80] | 0.84  [0.79 – 0.87] | 0.44  [0.35 – 0.53] | 0.94  [0.92 – 0.96] | 0.77  [0.67 – 0.84] | 0.37  [0.24 – 0.51] | 0.92  [0.87 – 0.96] |
| Barthel toilet use | 0.84  [0.79 – 0.87] | 0.74  [0.65 – 0.81] | 0.76  [0.72 – 0.80] | 0.76  [0.67 – 0.83] | 0.63  [0.49 – 0.76] | 0.74  [0.66 – 0.81] | 0.83  [0.79 – 0.87] | 0.46  [0.37 – 0.54] | 0.95  [0.92 – 0.97] | 0.76  [0.67 – 0.84] | 0.35  [0.22 – 0.49] | 0.93  [0.87 – 0.97] |
| Barthel | 0.84  [0.79 – 0.87] | 0.73  [0.64 – 0.80] | 0.78  [0.73 – 0.81] | 0.76  [0.67 – 0.83] | 0.59  [0.44 – 0.73] | 0.76  [0.68 – 0.83] | 0.84  [0.79 – 0.88] | 0.46  [0.38 – 0.55] | 0.95  [0.92 – 0.97] | 0.76  [0.68 – 0.84] | 0.39  [0.25 – 0.54] | 0.92  [0.87 – 0.96] |
| Myocardial infarction | 0.83  [0.79 – 0.87] | 0.74  [0.66 – 0.82] | 0.75  [0.71 – 0.79] | 0.76  [0.67 – 0.84] | 0.63  [0.49 – 0.76] | 0.74  [0.66 – 0.81] | 0.84  [0.79 – 0.87] | 0.44  [0.35 – 0.53] | 0.94  [0.92 – 0.96] | 0.76  [0.67 – 0.84] | 0.37  [0.24 – 0.51] | 0.92  [0.87 – 0.96] |
| Seizure | 0.84  [0.79 – 0.87] | 0.75  [0.67 – 0.82] | 0.76  [0.71 – 0.80] | 0.76  [0.67 – 0.84] | 0.61  [0.46 – 0.75] | 0.75  [0.67 – 0.82] | 0.83  [0.79 – 0.87] | 0.43  [0.35 – 0.52] | 0.94  [0.92 – 0.96] | 0.76  [0.67 – 0.84] | 0.39  [0.26 – 0.54] | 0.93  [0.87 – 0.97] |
| Alkohol abuse | 0.84  [0.79 – 0.88] | 0.76  [0.68 – 0.83] | 0.76  [0.72 – 0.80] | 0.76  [0.66 – 0.84] | 0.63  [0.48 – 0.77] | 0.75  [0.67 – 0.82] | 0.83  [0.78 – 0.86] | 0.42  [0.33 – 0.50] | 0.95  [0.92 – 0.97] | 0.77  [0.68 – 0.84] | 0.35  [0.22 – 0.49] | 0.93  [0.87 – 0.97] |
| Sex | 0.83  [0.79 – 0.87] | 0.74  [0.65 – 0.81] | 0.77  [0.73 – 0.81] | 0.76  [0.67 – 0.84] | 0.63  [0.49 – 0.76] | 0.75  [0.67 – 0.82] | 0.82  [0.77 – 0.86] | 0.42  [0.33 – 0.50] | 0.95  [0.93 – 0.97] | 0.77  [0.68 – 0.84] | 0.35  [0.22 – 0.49] | 0.93  [0.87 – 0.97] |
| Barthel stairs | 0.84  [0.79 – 0.87] | 0.76  [0.68 – 0.83] | 0.77  [0.72 – 0.81] | 0.76  [0.66 – 0.83] | 0.61  [0.46 – 0.75] | 0.75  [0.68 – 0.82] | 0.83  [0.79 – 0.87] | 0.43  [0.35 – 0.52] | 0.95  [0.92 – 0.96] | 0.76  [0.67 – 0.84] | 0.35  [0.22 – 0.49] | 0.93  [0.87 – 0.97] |
| Cerebrovascular disease | 0.84  [0.79 – 0.87] | 0.74  [0.66 – 0.82] | 0.78  [0.74 – 0.82] | 0.76  [0.67 – 0.83] | 0.61  [0.46 – 0.75] | 0.75  [0.67 – 0.82] | 0.83  [0.79 – 0.87] | 0.42  [0.34 – 0.51] | 0.95  [0.92 – 0.96] | 0.76  [0.67 – 0.84] | 0.35  [0.22 – 0.49] | 0.93  [0.87 – 0.97] |
| Parkinson’s disease | 0.84  [0.80 – 0.88] | 0.76  [0.68 – 0.83] | 0.77  [0.73 – 0.81] | 0.76  [0.66 – 0.83] | 0.61  [0.46 – 0.75] | 0.76  [0.68 – 0.83] | 0.83  [0.78 – 0.87] | 0.42  [0.33 – 0.50] | 0.95  [0.92 – 0.97] | 0.77  [0.68 – 0.84] | 0.35  [0.22 – 0.49] | 0.93  [0.87 – 0.97] |
| Barthelmobility | 0.84  [0.80 – 0.88] | 0.75  [0.67 – 0.82] | 0.76  [0.72 – 0.80] | 0.76  [0.66 – 0.83] | 0.63  [0.48 – 0.76] | 0.75  [0.67 – 0.82] | 0.83  [0.79 – 0.87] | 0.46  [0.37 – 0.54] | 0.94  [0.91 – 0.96] | 0.77  [0.67 – 0.84] | 0.35  [0.22 – 0.49] | 0.93  [0.87 – 0.97] |
| Number of children | 0.83  [0.79 – 0.87] | 0.77  [0.68 – 0.84] | 0.75  [0.71 – 0.79] | 0.76  [0.66 – 0.83] | 0.63  [0.49 – 0.76] | 0.77  [0.69 – 0.84] | 0.83  [0.78 – 0.86] | 0.45  [0.36 – 0.54] | 0.95  [0.92 – 0.97] | 0.76  [0.67 – 0.84] | 0.35  [0.22 – 0.49] | 0.93  [0.87 – 0.97] |
| Regional anesthesia | 0.84  [0.79 – 0.87] | 0.74  [0.65 – 0.81] | 0.77  [0.72 – 0.81] | 0.76  [0.66 – 0.83] | 0.63  [0.49 – 0.76] | 0.75  [0.67 – 0.82] | 0.83  [0.79 – 0.87] | 0.42  [0.34 – 0.51] | 0.94  [0.92 – 0.96] | 0.77  [0.67 – 0.84] | 0.35  [0.22 – 0.49] | 0.93  [0.87 – 0.97] |
| Has Pflegegrad | 0.83  [0.79 – 0.87] | 0.74  [0.66 – 0.81] | 0.76  [0.72 – 0.80] | 0.76  [0.66 – 0.83] | 0.63  [0.48 – 0.77] | 0.75  [0.67 – 0.82] | 0.83  [0.78 – 0.86] | 0.42  [0.33 – 0.50] | 0.95  [0.92 – 0.97] | 0.77  [0.68 – 0.84] | 0.35  [0.22 – 0.49] | 0.93  [0.87 – 0.97] |
| Creatinine | 0.84  [0.79 – 0.87] | 0.76  [0.68 – 0.83] | 0.75  [0.71 – 0.79] | 0.76  [0.66 – 0.83] | 0.63  [0.49 – 0.76] | 0.75  [0.67 – 0.82] | 0.83  [0.79 – 0.87] | 0.42  [0.34 – 0.51] | 0.94  [0.92 – 0.96] | 0.76  [0.67 – 0.84] | 0.37  [0.24 – 0.51] | 0.92  [0.87 – 0.96] |
| Living in a nursing home | 0.84  [0.79 – 0.87] | 0.74  [0.65 – 0.81] | 0.78  [0.73 – 0.81] | 0.76  [0.66 – 0.83] | 0.61  [0.46 – 0.75] | 0.76  [0.68 – 0.83] | 0.83  [0.78 – 0.87] | 0.42  [0.33 – 0.50] | 0.95  [0.92 – 0.97] | 0.77  [0.68 – 0.84] | 0.35  [0.22 – 0.49] | 0.93  [0.87 – 0.97] |
| Renal disease | 0.84  [0.79 – 0.87] | 0.74  [0.66 – 0.82] | 0.77  [0.73 – 0.81] | 0.76  [0.66 – 0.83] | 0.61  [0.46 – 0.75] | 0.76  [0.68 – 0.83] | 0.83  [0.79 – 0.87] | 0.42  [0.34 – 0.51] | 0.94  [0.92 – 0.96] | 0.76  [0.67 – 0.84] | 0.37  [0.24 – 0.51] | 0.93  [0.87 – 0.97] |
| Barthel feeding | 0.84  [0.79 – 0.88] | 0.75  [0.67 – 0.82] | 0.76  [0.72 – 0.80] | 0.76  [0.66 – 0.83] | 0.61  [0.46 – 0.75] | 0.75  [0.67 – 0.82] | 0.83  [0.78 – 0.86] | 0.42  [0.34 – 0.51] | 0.95  [0.93 – 0.97] | 0.76  [0.67 – 0.84] | 0.33  [0.20 – 0.47] | 0.93  [0.87 – 0.97] |
| PHQ-4 anxiety | 0.84  [0.79 – 0.87] | 0.77  [0.69 – 0.84] | 0.77  [0.73 – 0.81] | 0.76  [0.66 – 0.83] | 0.63  [0.48 – 0.76] | 0.75  [0.67 – 0.82] | 0.83  [0.78 – 0.87] | 0.39  [0.31 – 0.48] | 0.95  [0.92 – 0.96] | 0.77  [0.68 – 0.84] | 0.35  [0.22 – 0.49] | 0.95  [0.89 – 0.98] |
| Height | 0.84  [0.79 – 0.87] | 0.73  [0.64 – 0.80] | 0.77  [0.73 – 0.81] | 0.76  [0.66 – 0.83] | 0.61  [0.46 – 0.75] | 0.76  [0.68 – 0.83] | 0.83  [0.79 – 0.87] | 0.42  [0.34 – 0.51] | 0.94  [0.92 – 0.96] | 0.76  [0.67 – 0.84] | 0.35  [0.22 – 0.49] | 0.93  [0.87 – 0.97] |
| Hearing aid | 0.84  [0.79 – 0.87] | 0.74  [0.66 – 0.81] | 0.76  [0.72 – 0.80] | 0.76  [0.66 – 0.83] | 0.63  [0.49 – 0.76] | 0.75  [0.67 – 0.82] | 0.83  [0.78 – 0.87] | 0.42  [0.34 – 0.51] | 0.95  [0.92 – 0.97] | 0.77  [0.68 – 0.84] | 0.35  [0.22 – 0.49] | 0.93  [0.87 – 0.97] |
| Digitspan backwards test | 0.84  [0.79 – 0.88] | 0.76  [0.68 – 0.83] | 0.75  [0.71 – 0.79] | 0.76  [0.66 – 0.83] | 0.65  [0.51 – 0.78] | 0.75  [0.67 – 0.82] | 0.83  [0.79 – 0.87] | 0.43  [0.35 – 0.52] | 0.95  [0.92 – 0.96] | 0.76  [0.67 – 0.84] | 0.37  [0.24 – 0.51] | 0.93  [0.87 – 0.97] |
| Living with relatives | 0.84  [0.79 – 0.87] | 0.75  [0.67 – 0.82] | 0.76  [0.71 – 0.80] | 0.76  [0.66 – 0.83] | 0.63  [0.49 – 0.76] | 0.76  [0.68 – 0.83] | 0.82  [0.77 – 0.86] | 0.39  [0.31 – 0.48] | 0.96  [0.94 – 0.98] | 0.76  [0.67 – 0.84] | 0.26  [0.15 – 0.40] | 0.96  [0.92 – 0.99] |
| Surgery setting visceral | 0.84  [0.79 – 0.87] | 0.73  [0.64 – 0.80] | 0.77  [0.73 – 0.81] | 0.76  [0.66 – 0.83] | 0.61  [0.46 – 0.75] | 0.75  [0.67 – 0.82] | 0.83  [0.78 – 0.86] | 0.42  [0.33 – 0.50] | 0.95  [0.92 – 0.97] | 0.77  [0.68 – 0.84] | 0.35  [0.22 – 0.49] | 0.93  [0.87 – 0.97] |
| Weight | 0.83  [0.79 – 0.87] | 0.74  [0.66 – 0.82] | 0.75  [0.71 – 0.79] | 0.76  [0.66 – 0.83] | 0.63  [0.48 – 0.76] | 0.75  [0.67 – 0.82] | 0.83  [0.78 – 0.86] | 0.42  [0.34 – 0.51] | 0.95  [0.93 – 0.97] | 0.77  [0.68 – 0.84] | 0.35  [0.22 – 0.49] | 0.93  [0.87 – 0.97] |
| Other liver disease | 0.84  [0.79 – 0.87] | 0.74  [0.66 – 0.82] | 0.76  [0.72 – 0.80] | 0.76  [0.66 – 0.83] | 0.61  [0.46 – 0.75] | 0.75  [0.67 – 0.82] | 0.83  [0.79 – 0.87] | 0.42  [0.34 – 0.51] | 0.94  [0.92 – 0.96] | 0.77  [0.67 – 0.84] | 0.35  [0.22 – 0.49] | 0.93  [0.87 – 0.97] |
| Pre-operative opioids | 0.84  [0.79 – 0.87] | 0.74  [0.66 – 0.82] | 0.76  [0.71 – 0.80] | 0.75  [0.66 – 0.83] | 0.61  [0.46 – 0.75] | 0.75  [0.67 – 0.82] | 0.83  [0.78 – 0.87] | 0.42  [0.34 – 0.51] | 0.95  [0.92 – 0.97] | 0.77  [0.67 – 0.84] | 0.35  [0.22 – 0.49] | 0.93  [0.87 – 0.97] |
| PHQ-4 worrying | 0.84  [0.79 – 0.87] | 0.74  [0.66 – 0.81] | 0.76  [0.72 – 0.80] | 0.75  [0.66 – 0.83] | 0.61  [0.46 – 0.75] | 0.74  [0.66 – 0.81] | 0.83  [0.79 – 0.87] | 0.43  [0.35 – 0.52] | 0.94  [0.92 – 0.96] | 0.77  [0.67 – 0.84] | 0.35  [0.22 – 0.49] | 0.95  [0.89 – 0.98] |
| Vascular disease | 0.84  [0.79 – 0.87] | 0.75  [0.67 – 0.82] | 0.77  [0.72 – 0.81] | 0.75  [0.66 – 0.83] | 0.61  [0.46 – 0.75] | 0.74  [0.66 – 0.81] | 0.83  [0.79 – 0.87] | 0.42  [0.34 – 0.51] | 0.95  [0.92 – 0.96] | 0.76  [0.67 – 0.84] | 0.35  [0.22 – 0.49] | 0.93  [0.87 – 0.97] |
| Nicotine abuse | 0.84  [0.79 – 0.87] | 0.73  [0.64 – 0.80] | 0.77  [0.72 – 0.81] | 0.75  [0.66 – 0.83] | 0.63  [0.49 – 0.76] | 0.74  [0.66 – 0.81] | 0.83  [0.78 – 0.87] | 0.42  [0.33 – 0.50] | 0.95  [0.92 – 0.97] | 0.77  [0.68 – 0.84] | 0.35  [0.22 – 0.49] | 0.93  [0.87 – 0.97] |
| Divorced | 0.84  [0.79 – 0.87] | 0.75  [0.67 – 0.82] | 0.76  [0.72 – 0.80] | 0.75  [0.66 – 0.83] | 0.61  [0.46 – 0.75] | 0.75  [0.67 – 0.82] | 0.83  [0.78 – 0.86] | 0.42  [0.33 – 0.50] | 0.95  [0.92 – 0.97] | 0.77  [0.68 – 0.84] | 0.35  [0.22 – 0.49] | 0.93  [0.87 – 0.97] |
| Level of Pflegegrad | 0.84  [0.79 – 0.87] | 0.75  [0.67 – 0.82] | 0.77  [0.73 – 0.81] | 0.75  [0.66 – 0.83] | 0.63  [0.48 – 0.76] | 0.76  [0.68 – 0.83] | 0.83  [0.79 – 0.87] | 0.42  [0.34 – 0.51] | 0.95  [0.92 – 0.96] | 0.77  [0.67 – 0.84] | 0.35  [0.22 – 0.49] | 0.93  [0.87 – 0.97] |
| Widowed | 0.84  [0.79 – 0.87] | 0.74  [0.66 – 0.81] | 0.75  [0.70 – 0.79] | 0.75  [0.66 – 0.83] | 0.61  [0.46 – 0.75] | 0.74  [0.66 – 0.81] | 0.83  [0.79 – 0.87] | 0.42  [0.34 – 0.51] | 0.95  [0.92 – 0.97] | 0.76  [0.67 – 0.84] | 0.35  [0.22 – 0.49] | 0.93  [0.87 – 0.97] |
| Prior delirium | 0.84  [0.79 – 0.87] | 0.75  [0.67 – 0.82] | 0.77  [0.73 – 0.81] | 0.75  [0.66 – 0.83] | 0.61  [0.46 – 0.75] | 0.75  [0.67 – 0.82] | 0.83  [0.79 – 0.87] | 0.44  [0.35 – 0.53] | 0.95  [0.92 – 0.97] | 0.76  [0.67 – 0.84] | 0.35  [0.22 – 0.49] | 0.93  [0.87 – 0.97] |
| Lost more than 3 kg | 0.84  [0.79 – 0.87] | 0.75  [0.67 – 0.82] | 0.77  [0.72 – 0.81] | 0.75  [0.66 – 0.83] | 0.61  [0.46 – 0.75] | 0.75  [0.67 – 0.82] | 0.83  [0.79 – 0.87] | 0.43  [0.34 – 0.52] | 0.94  [0.92 – 0.96] | 0.76  [0.67 – 0.84] | 0.35  [0.22 – 0.49] | 0.93  [0.87 – 0.97] |
| Source of anamnesis: realtives | 0.84  [0.79 – 0.88] | 0.77  [0.69 – 0.83] | 0.75  [0.71 – 0.79] | 0.75  [0.66 – 0.83] | 0.63  [0.49 – 0.76] | 0.75  [0.67 – 0.82] | 0.83  [0.78 – 0.87] | 0.42  [0.33 – 0.50] | 0.95  [0.92 – 0.97] | 0.77  [0.67 – 0.84] | 0.35  [0.22 – 0.49] | 0.93  [0.87 – 0.97] |
| Arthrosis of the hand | 0.84  [0.79 – 0.87] | 0.74  [0.65 – 0.81] | 0.76  [0.72 – 0.80] | 0.75  [0.66 – 0.83] | 0.61  [0.46 – 0.75] | 0.74  [0.66 – 0.81] | 0.83  [0.79 – 0.87] | 0.42  [0.34 – 0.51] | 0.94  [0.92 – 0.96] | 0.76  [0.67 – 0.84] | 0.35  [0.22 – 0.49] | 0.93  [0.87 – 0.97] |
| Lost 1–3 kg | 0.84  [0.79 – 0.87] | 0.74  [0.66 – 0.82] | 0.76  [0.72 – 0.80] | 0.75  [0.66 – 0.83] | 0.61  [0.46 – 0.75] | 0.75  [0.67 – 0.82] | 0.83  [0.79 – 0.87] | 0.42  [0.34 – 0.51] | 0.95  [0.92 – 0.96] | 0.76  [0.67 – 0.84] | 0.35  [0.22 – 0.49] | 0.93  [0.87 – 0.97] |
| Surgery type: urogenital | 0.84  [0.79 – 0.87] | 0.76  [0.68 – 0.83] | 0.77  [0.72 – 0.81] | 0.75  [0.66 – 0.83] | 0.61  [0.46 – 0.75] | 0.75  [0.67 – 0.82] | 0.83  [0.78 – 0.86] | 0.42  [0.33 – 0.50] | 0.95  [0.92 – 0.97] | 0.77  [0.68 – 0.84] | 0.35  [0.22 – 0.49] | 0.93  [0.87 – 0.97] |
| Surgery type: abdominal | 0.84  [0.79 – 0.87] | 0.74  [0.65 – 0.81] | 0.77  [0.72 – 0.81] | 0.75  [0.66 – 0.83] | 0.61  [0.46 – 0.75] | 0.75  [0.67 – 0.82] | 0.83  [0.78 – 0.87] | 0.42  [0.33 – 0.50] | 0.95  [0.92 – 0.97] | 0.77  [0.67 – 0.84] | 0.35  [0.22 – 0.49] | 0.93  [0.87 – 0.97] |
| Heamoglobin | 0.84  [0.79 – 0.87] | 0.74  [0.66 – 0.81] | 0.77  [0.73 – 0.81] | 0.75  [0.66 – 0.83] | 0.61  [0.46 – 0.75] | 0.76  [0.68 – 0.83] | 0.83  [0.79 – 0.87] | 0.42  [0.34 – 0.51] | 0.94  [0.92 – 0.96] | 0.76  [0.67 – 0.84] | 0.35  [0.22 – 0.49] | 0.92  [0.87 – 0.96] |
| Pain | 0.83  [0.79 – 0.87] | 0.74  [0.65 – 0.81] | 0.76  [0.72 – 0.80] | 0.75  [0.66 – 0.83] | 0.61  [0.46 – 0.75] | 0.73  [0.65 – 0.80] | 0.83  [0.78 – 0.87] | 0.43  [0.35 – 0.52] | 0.95  [0.92 – 0.97] | 0.76  [0.67 – 0.84] | 0.35  [0.22 – 0.49] | 0.93  [0.87 – 0.97] |
| EQ-5D-5L Anxiety | 0.84  [0.79 – 0.87] | 0.74  [0.66 – 0.82] | 0.77  [0.73 – 0.81] | 0.75  [0.66 – 0.83] | 0.61  [0.46 – 0.75] | 0.77  [0.69 – 0.84] | 0.83  [0.78 – 0.87] | 0.43  [0.35 – 0.52] | 0.95  [0.93 – 0.97] | 0.77  [0.67 – 0.84] | 0.35  [0.22 – 0.49] | 0.93  [0.87 – 0.97] |
| Living with spouse | 0.83  [0.79 – 0.87] | 0.74  [0.66 – 0.82] | 0.76  [0.72 – 0.80] | 0.75  [0.66 – 0.83] | 0.61  [0.46 – 0.75] | 0.78  [0.70 – 0.84] | 0.81  [0.77 – 0.85] | 0.39  [0.31 – 0.48] | 0.96  [0.94 – 0.98] | 0.77  [0.67 – 0.84] | 0.26  [0.15 – 0.40] | 0.96  [0.92 – 0.99] |
| Married | 0.84  [0.79 – 0.87] | 0.74  [0.66 – 0.81] | 0.76  [0.71 – 0.80] | 0.75  [0.66 – 0.83] | 0.61  [0.46 – 0.75] | 0.74  [0.66 – 0.81] | 0.83  [0.79 – 0.87] | 0.42  [0.34 – 0.51] | 0.94  [0.92 – 0.96] | 0.76  [0.67 – 0.84] | 0.35  [0.22 – 0.49] | 0.93  [0.87 – 0.97] |
| Amount of recent falls | 0.84  [0.79 – 0.87] | 0.74  [0.66 – 0.82] | 0.76  [0.72 – 0.80] | 0.75  [0.66 – 0.83] | 0.61  [0.46 – 0.75] | 0.75  [0.67 – 0.82] | 0.84  [0.79 – 0.87] | 0.43  [0.35 – 0.52] | 0.95  [0.92 – 0.96] | 0.77  [0.67 – 0.84] | 0.35  [0.22 – 0.49] | 0.93  [0.87 – 0.97] |
| Neurological condition | 0.84  [0.79 – 0.87] | 0.75  [0.67 – 0.82] | 0.74  [0.69 – 0.78] | 0.75  [0.66 – 0.83] | 0.61  [0.46 – 0.75] | 0.75  [0.67 – 0.82] | 0.83  [0.78 – 0.87] | 0.43  [0.35 – 0.52] | 0.94  [0.92 – 0.96] | 0.76  [0.67 – 0.84] | 0.35  [0.22 – 0.49] | 0.93  [0.87 – 0.97] |
| Living in retirement home | 0.84  [0.79 – 0.88] | 0.76  [0.68 – 0.83] | 0.78  [0.73 – 0.82] | 0.75  [0.66 – 0.83] | 0.61  [0.46 – 0.75] | 0.76  [0.68 – 0.83] | 0.83  [0.78 – 0.86] | 0.42  [0.33 – 0.50] | 0.95  [0.92 – 0.97] | 0.77  [0.68 – 0.84] | 0.35  [0.22 – 0.49] | 0.93  [0.87 – 0.97] |
| Cancer | 0.83  [0.79 – 0.87] | 0.78  [0.70 – 0.84] | 0.74  [0.69 – 0.78] | 0.75  [0.66 – 0.83] | 0.63  [0.48 – 0.76] | 0.72  [0.64 – 0.79] | 0.83  [0.79 – 0.87] | 0.42  [0.34 – 0.51] | 0.95  [0.92 – 0.96] | 0.76  [0.67 – 0.84] | 0.35  [0.22 – 0.49] | 0.93  [0.87 – 0.97] |
| Surgery type: lungs | 0.84  [0.79 – 0.87] | 0.74  [0.66 – 0.82] | 0.77  [0.72 – 0.81] | 0.75  [0.66 – 0.83] | 0.61  [0.46 – 0.75] | 0.75  [0.67 – 0.82] | 0.83  [0.78 – 0.86] | 0.42  [0.33 – 0.50] | 0.95  [0.92 – 0.97] | 0.77  [0.68 – 0.84] | 0.35  [0.22 – 0.49] | 0.93  [0.87 – 0.97] |
| Spinal anesthesia | 0.84  [0.79 – 0.87] | 0.75  [0.67 – 0.82] | 0.77  [0.72 – 0.81] | 0.75  [0.66 – 0.83] | 0.61  [0.46 – 0.75] | 0.75  [0.67 – 0.82] | 0.83  [0.78 – 0.86] | 0.42  [0.33 – 0.50] | 0.95  [0.92 – 0.97] | 0.77  [0.68 – 0.84] | 0.35  [0.22 – 0.49] | 0.93  [0.87 – 0.97] |
| Source of anamnesis: other | 0.84  [0.79 – 0.87] | 0.74  [0.66 – 0.82] | 0.77  [0.72 – 0.81] | 0.75  [0.66 – 0.83] | 0.61  [0.46 – 0.75] | 0.75  [0.67 – 0.82] | 0.83  [0.78 – 0.86] | 0.42  [0.33 – 0.50] | 0.95  [0.92 – 0.97] | 0.77  [0.68 – 0.84] | 0.35  [0.22 – 0.49] | 0.93  [0.87 – 0.97] |
| **Base 15-feature model** | **0.84**  **[0.79 – 0.87]** | **0.74**  **[0.66 – 0.82]** | **0.77**  **[0.72 – 0.81]** | **0.75**  **[0.66 – 0.83]** | **0.61**  **[0.46 – 0.75]** | **0.75**  **[0.67 – 0.82]** | **0.83**  **[0.78 – 0.86]** | **0.42**  **[0.33 – 0.50]** | **0.95**  **[0.92 – 0.97]** | **0.77**  **[0.68 – 0.84]** | **0.35**  **[0.22 – 0.49]** | **0.93**  **[0.87 – 0.97]** |
| Diabetes without complications | 0.84  [0.79 – 0.87] | 0.75  [0.67 – 0.82] | 0.77  [0.72 – 0.81] | 0.75  [0.66 – 0.83] | 0.61  [0.46 – 0.75] | 0.74  [0.66 – 0.81] | 0.84  [0.79 – 0.87] | 0.44  [0.35 – 0.53] | 0.94  [0.92 – 0.96] | 0.76  [0.67 – 0.84] | 0.37  [0.24 – 0.51] | 0.92  [0.87 – 0.96] |
| Surgery setting: general | 0.84  [0.79 – 0.87] | 0.74  [0.65 – 0.81] | 0.77  [0.73 – 0.81] | 0.75  [0.66 – 0.83] | 0.61  [0.46 – 0.75] | 0.76  [0.68 – 0.83] | 0.83  [0.78 – 0.86] | 0.42  [0.33 – 0.50] | 0.95  [0.92 – 0.97] | 0.77  [0.68 – 0.84] | 0.35  [0.22 – 0.49] | 0.93  [0.87 – 0.97] |
| Mobile outside | 0.84  [0.79 – 0.87] | 0.75  [0.67 – 0.82] | 0.77  [0.72 – 0.81] | 0.75  [0.66 – 0.83] | 0.61  [0.46 – 0.75] | 0.75  [0.67 – 0.82] | 0.84  [0.79 – 0.87] | 0.43  [0.34 – 0.52] | 0.94  [0.92 – 0.96] | 0.76  [0.67 – 0.84] | 0.35  [0.22 – 0.49] | 0.93  [0.87 – 0.97] |
| Congestive heart failure | 0.84  [0.79 – 0.87] | 0.75  [0.67 – 0.82] | 0.76  [0.72 – 0.80] | 0.75  [0.66 – 0.83] | 0.61  [0.46 – 0.75] | 0.75  [0.67 – 0.82] | 0.83  [0.79 – 0.87] | 0.42  [0.34 – 0.51] | 0.94  [0.92 – 0.96] | 0.76  [0.67 – 0.84] | 0.35  [0.22 – 0.49] | 0.93  [0.87 – 0.97] |
| PHQ-4 feeling down | 0.84  [0.79 – 0.87] | 0.75  [0.67 – 0.82] | 0.76  [0.72 – 0.80] | 0.75  [0.66 – 0.83] | 0.61  [0.46 – 0.75] | 0.75  [0.67 – 0.82] | 0.83  [0.78 – 0.87] | 0.42  [0.33 – 0.50] | 0.95  [0.92 – 0.96] | 0.77  [0.67 – 0.84] | 0.35  [0.22 – 0.49] | 0.93  [0.87 – 0.97] |
| Lost less than 1 kg | 0.83  [0.79 – 0.87] | 0.75  [0.67 – 0.82] | 0.75  [0.70 – 0.79] | 0.75  [0.66 – 0.83] | 0.63  [0.48 – 0.77] | 0.75  [0.67 – 0.81] | 0.81  [0.77 – 0.85] | 0.41  [0.32 – 0.50] | 0.95  [0.92 – 0.96] | 0.77  [0.68 – 0.84] | 0.28  [0.16 – 0.43] | 0.95  [0.90 – 0.98] |
| Other arthrosis | 0.84  [0.79 – 0.87] | 0.74  [0.66 – 0.81] | 0.79  [0.75 – 0.83] | 0.75  [0.66 – 0.83] | 0.63  [0.49 – 0.76] | 0.76  [0.68 – 0.83] | 0.83  [0.79 – 0.87] | 0.46  [0.37 – 0.55] | 0.94  [0.91 – 0.96] | 0.76  [0.67 – 0.84] | 0.37  [0.24 – 0.51] | 0.93  [0.87 – 0.97] |
| EQ-5D-5L usual activities | 0.84  [0.79 – 0.87] | 0.76  [0.68 – 0.83] | 0.76  [0.71 – 0.80] | 0.75  [0.66 – 0.83] | 0.63  [0.49 – 0.76] | 0.74  [0.66 – 0.81] | 0.82  [0.78 – 0.86] | 0.42  [0.33 – 0.50] | 0.94  [0.92 – 0.96] | 0.76  [0.67 – 0.84] | 0.35  [0.22 – 0.49] | 0.93  [0.87 – 0.97] |
| Barthel bowel control | 0.84  [0.79 – 0.87] | 0.75  [0.67 – 0.82] | 0.77  [0.72 – 0.81] | 0.75  [0.66 – 0.83] | 0.61  [0.46 – 0.75] | 0.75  [0.67 – 0.82] | 0.83  [0.78 – 0.87] | 0.43  [0.35 – 0.52] | 0.95  [0.92 – 0.96] | 0.76  [0.67 – 0.84] | 0.35  [0.22 – 0.49] | 0.93  [0.87 – 0.97] |
| EQ-5D-5L visual analog scale | 0.84  [0.79 – 0.87] | 0.75  [0.67 – 0.82] | 0.77  [0.72 – 0.81] | 0.75  [0.66 – 0.83] | 0.61  [0.46 – 0.75] | 0.75  [0.67 – 0.82] | 0.84  [0.79 – 0.87] | 0.44  [0.35 – 0.53] | 0.94  [0.92 – 0.96] | 0.76  [0.67 – 0.84] | 0.35  [0.22 – 0.49] | 0.92  [0.87 – 0.96] |
| Anesthesia with intubation | 0.84  [0.79 – 0.87] | 0.75  [0.67 – 0.82] | 0.77  [0.72 – 0.81] | 0.75  [0.66 – 0.83] | 0.61  [0.46 – 0.75] | 0.75  [0.67 – 0.82] | 0.83  [0.78 – 0.87] | 0.44  [0.36 – 0.53] | 0.95  [0.92 – 0.96] | 0.76  [0.67 – 0.84] | 0.35  [0.22 – 0.49] | 0.93  [0.87 – 0.97] |
| Pre-operative antipsychotics | 0.84  [0.80 – 0.88] | 0.75  [0.67 – 0.82] | 0.78  [0.73 – 0.81] | 0.75  [0.66 – 0.83] | 0.61  [0.46 – 0.75] | 0.75  [0.67 – 0.82] | 0.84  [0.79 – 0.87] | 0.44  [0.35 – 0.53] | 0.95  [0.92 – 0.97] | 0.76  [0.67 – 0.84] | 0.35  [0.22 – 0.49] | 0.93  [0.87 – 0.97] |
| Mobile at home | 0.84  [0.79 – 0.87] | 0.74  [0.66 – 0.82] | 0.77  [0.73 – 0.81] | 0.75  [0.66 – 0.83] | 0.61  [0.46 – 0.75] | 0.75  [0.67 – 0.82] | 0.83  [0.79 – 0.87] | 0.42  [0.34 – 0.51] | 0.94  [0.91 – 0.96] | 0.76  [0.67 – 0.84] | 0.37  [0.24 – 0.51] | 0.92  [0.87 – 0.96] |
| Bad memory | 0.84  [0.79 – 0.87] | 0.78  [0.69 – 0.84] | 0.75  [0.70 – 0.79] | 0.75  [0.65 – 0.83] | 0.65  [0.51 – 0.78] | 0.75  [0.67 – 0.82] | 0.83  [0.78 – 0.86] | 0.42  [0.34 – 0.51] | 0.95  [0.92 – 0.97] | 0.77  [0.68 – 0.84] | 0.35  [0.22 – 0.49] | 0.93  [0.87 – 0.97] |
| Diabetes, any kind | 0.83  [0.79 – 0.87] | 0.75  [0.67 – 0.82] | 0.77  [0.72 – 0.81] | 0.75  [0.66 – 0.83] | 0.61  [0.46 – 0.75] | 0.74  [0.66 – 0.81] | 0.83  [0.78 – 0.87] | 0.42  [0.34 – 0.51] | 0.95  [0.92 – 0.96] | 0.77  [0.68 – 0.84] | 0.35  [0.22 – 0.49] | 0.93  [0.87 – 0.97] |
| Has children | 0.84  [0.79 – 0.88] | 0.76  [0.68 – 0.83] | 0.75  [0.71 – 0.79] | 0.75  [0.66 – 0.83] | 0.61  [0.46 – 0.75] | 0.76  [0.68 – 0.83] | 0.82  [0.77 – 0.86] | 0.38  [0.30 – 0.47] | 0.97  [0.95 – 0.98] | 0.76  [0.67 – 0.84] | 0.28  [0.16 – 0.42] | 0.96  [0.92 – 0.99] |
| Barthel grooming | 0.84  [0.79 – 0.87] | 0.75  [0.67 – 0.82] | 0.78  [0.73 – 0.82] | 0.75  [0.66 – 0.83] | 0.61  [0.46 – 0.75] | 0.76  [0.68 – 0.83] | 0.83  [0.79 – 0.87] | 0.42  [0.34 – 0.51] | 0.94  [0.92 – 0.96] | 0.77  [0.67 – 0.84] | 0.35  [0.22 – 0.49] | 0.93  [0.87 – 0.97] |
| Anesthesia with larynx mask | 0.84  [0.79 – 0.87] | 0.76  [0.68 – 0.83] | 0.76  [0.72 – 0.80] | 0.75  [0.66 – 0.83] | 0.61  [0.46 – 0.75] | 0.75  [0.67 – 0.82] | 0.83  [0.79 – 0.87] | 0.42  [0.34 – 0.51] | 0.94  [0.91 – 0.96] | 0.76  [0.67 – 0.84] | 0.37  [0.24 – 0.51] | 0.93  [0.87 – 0.97] |
| EQ-5D-5L mobility | 0.84  [0.79 – 0.88] | 0.75  [0.67 – 0.82] | 0.76  [0.71 – 0.80] | 0.75  [0.66 – 0.83] | 0.61  [0.46 – 0.75] | 0.74  [0.66 – 0.81] | 0.84  [0.79 – 0.87] | 0.45  [0.36 – 0.53] | 0.95  [0.92 – 0.97] | 0.75  [0.66 – 0.83] | 0.37  [0.24 – 0.51] | 0.92  [0.87 – 0.96] |
| Stroke | 0.84  [0.79 – 0.87] | 0.78  [0.71 – 0.85] | 0.75  [0.71 – 0.79] | 0.75  [0.66 – 0.83] | 0.61  [0.46 – 0.75] | 0.74  [0.66 – 0.81] | 0.83  [0.79 – 0.87] | 0.42  [0.34 – 0.51] | 0.95  [0.92 – 0.96] | 0.76  [0.67 – 0.84] | 0.35  [0.22 – 0.49] | 0.93  [0.87 – 0.97] |
| Sensory deprivation | 0.84  [0.79 – 0.87] | 0.74  [0.66 – 0.82] | 0.77  [0.72 – 0.81] | 0.75  [0.66 – 0.83] | 0.63  [0.49 – 0.76] | 0.77  [0.69 – 0.84] | 0.81  [0.77 – 0.85] | 0.37  [0.29 – 0.46] | 0.96  [0.94 – 0.98] | 0.76  [0.67 – 0.84] | 0.30  [0.18 – 0.45] | 0.94  [0.89 – 0.97] |
| Body mass index | 0.83  [0.79 – 0.87] | 0.74  [0.65 – 0.81] | 0.76  [0.71 – 0.80] | 0.75  [0.66 – 0.83] | 0.63  [0.48 – 0.76] | 0.75  [0.67 – 0.82] | 0.83  [0.79 – 0.87] | 0.43  [0.35 – 0.52] | 0.95  [0.92 – 0.97] | 0.76  [0.67 – 0.84] | 0.35  [0.22 – 0.49] | 0.92  [0.87 – 0.96] |
| Liver disease, any kind | 0.83  [0.79 – 0.87] | 0.75  [0.67 – 0.82] | 0.77  [0.73 – 0.81] | 0.75  [0.66 – 0.83] | 0.61  [0.46 – 0.75] | 0.75  [0.67 – 0.82] | 0.83  [0.78 – 0.87] | 0.42  [0.34 – 0.51] | 0.95  [0.92 – 0.96] | 0.76  [0.67 – 0.83] | 0.35  [0.22 – 0.49] | 0.93  [0.87 – 0.97] |
| Living with other family members | 0.84  [0.79 – 0.87] | 0.76  [0.68 – 0.83] | 0.77  [0.72 – 0.81] | 0.75  [0.66 – 0.83] | 0.61  [0.46 – 0.75] | 0.75  [0.67 – 0.82] | 0.83  [0.78 – 0.86] | 0.42  [0.33 – 0.50] | 0.95  [0.92 – 0.97] | 0.77  [0.68 – 0.84] | 0.35  [0.22 – 0.49] | 0.93  [0.87 – 0.97] |
| Barthel bladder control | 0.84  [0.79 – 0.87] | 0.75  [0.67 – 0.82] | 0.76  [0.72 – 0.80] | 0.75  [0.66 – 0.83] | 0.63  [0.48 – 0.77] | 0.75  [0.67 – 0.82] | 0.83  [0.79 – 0.87] | 0.43  [0.35 – 0.52] | 0.94  [0.92 – 0.96] | 0.76  [0.67 – 0.84] | 0.35  [0.22 – 0.49] | 0.93  [0.87 – 0.97] |
| Time under general anesthesia | 0.84  [0.79 – 0.88] | 0.74  [0.66 – 0.82] | 0.77  [0.72 – 0.81] | 0.75  [0.66 – 0.83] | 0.61  [0.46 – 0.75] | 0.75  [0.66 – 0.82] | 0.83  [0.79 – 0.87] | 0.42  [0.34 – 0.51] | 0.94  [0.92 – 0.96] | 0.76  [0.67 – 0.84] | 0.35  [0.22 – 0.49] | 0.93  [0.87 – 0.97] |
| EQ-5D-5L self-care | 0.84  [0.79 – 0.88] | 0.74  [0.65 – 0.81] | 0.79  [0.74 – 0.83] | 0.75  [0.65 – 0.83] | 0.65  [0.51 – 0.79] | 0.75  [0.66 – 0.81] | 0.84  [0.79 – 0.88] | 0.49  [0.40 – 0.57] | 0.95  [0.92 – 0.96] | 0.76  [0.66 – 0.83] | 0.41  [0.27 – 0.56] | 0.92  [0.87 – 0.96] |
| PHQ-4 nervous | 0.84  [0.79 – 0.87] | 0.74  [0.66 – 0.82] | 0.76  [0.71 – 0.80] | 0.75  [0.66 – 0.83] | 0.63  [0.49 – 0.76] | 0.74  [0.66 – 0.81] | 0.83  [0.78 – 0.87] | 0.42  [0.34 – 0.51] | 0.95  [0.92 – 0.96] | 0.76  [0.67 – 0.84] | 0.37  [0.24 – 0.51] | 0.94  [0.88 – 0.97] |
| Source of anamnesis: chart | 0.84  [0.80 – 0.88] | 0.76  [0.68 – 0.83] | 0.78  [0.74 – 0.82] | 0.75  [0.65 – 0.83] | 0.63  [0.48 – 0.76] | 0.73  [0.65 – 0.80] | 0.84  [0.79 – 0.88] | 0.41  [0.33 – 0.50] | 0.94  [0.92 – 0.96] | 0.76  [0.67 – 0.84] | 0.33  [0.20 – 0.47] | 0.95  [0.89 – 0.98] |
| Surgery setting: orthopedic | 0.84  [0.79 – 0.87] | 0.75  [0.67 – 0.82] | 0.75  [0.71 – 0.79] | 0.75  [0.65 – 0.83] | 0.65  [0.51 – 0.78] | 0.74  [0.66 – 0.81] | 0.83  [0.78 – 0.87] | 0.43  [0.35 – 0.52] | 0.95  [0.92 – 0.97] | 0.76  [0.67 – 0.84] | 0.35  [0.22 – 0.49] | 0.93  [0.87 – 0.97] |
| Source of anamnesis: patient | 0.84  [0.79 – 0.88] | 0.76  [0.68 – 0.83] | 0.75  [0.71 – 0.79] | 0.75  [0.66 – 0.83] | 0.61  [0.46 – 0.74] | 0.73  [0.65 – 0.80] | 0.81  [0.76 – 0.85] | 0.39  [0.31 – 0.48] | 0.95  [0.92 – 0.97] | 0.76  [0.68 – 0.84] | 0.33  [0.20 – 0.47] | 0.92  [0.87 – 0.96] |
| General anesthesion using IVs | 0.84  [0.79 – 0.88] | 0.76  [0.68 – 0.83] | 0.78  [0.74 – 0.82] | 0.75  [0.66 – 0.83] | 0.61  [0.46 – 0.75] | 0.73  [0.65 – 0.80] | 0.82  [0.78 – 0.86] | 0.41  [0.33 – 0.50] | 0.96  [0.93 – 0.98] | 0.75  [0.66 – 0.83] | 0.33  [0.20 – 0.47] | 0.93  [0.87 – 0.97] |
| Diabetes with complications | 0.84  [0.79 – 0.87] | 0.76  [0.68 – 0.83] | 0.77  [0.72 – 0.81] | 0.75  [0.65 – 0.83] | 0.63  [0.49 – 0.76] | 0.75  [0.67 – 0.82] | 0.83  [0.79 – 0.87] | 0.44  [0.35 – 0.53] | 0.94  [0.91 – 0.96] | 0.76  [0.67 – 0.84] | 0.35  [0.22 – 0.49] | 0.93  [0.87 – 0.97] |
| Separated from spouse | 0.84  [0.79 – 0.88] | 0.75  [0.67 – 0.82] | 0.76  [0.71 – 0.80] | 0.75  [0.65 – 0.83] | 0.63  [0.49 – 0.76] | 0.75  [0.67 – 0.82] | 0.83  [0.78 – 0.87] | 0.42  [0.33 – 0.50] | 0.95  [0.92 – 0.96] | 0.77  [0.68 – 0.84] | 0.35  [0.22 – 0.49] | 0.93  [0.87 – 0.97] |
| Hypotension | 0.84  [0.80 – 0.88] | 0.77  [0.69 – 0.83] | 0.76  [0.72 – 0.80] | 0.75  [0.65 – 0.83] | 0.63  [0.49 – 0.76] | 0.72  [0.63 – 0.79] | 0.83  [0.78 – 0.87] | 0.43  [0.35 – 0.52] | 0.95  [0.92 – 0.96] | 0.77  [0.68 – 0.84] | 0.35  [0.22 – 0.49] | 0.93  [0.87 – 0.97] |
| Pflegegrad is requested | 0.84  [0.79 – 0.88] | 0.76  [0.68 – 0.83] | 0.76  [0.72 – 0.80] | 0.75  [0.66 – 0.83] | 0.57  [0.42 – 0.70] | 0.76  [0.68 – 0.83] | 0.83  [0.79 – 0.87] | 0.42  [0.34 – 0.51] | 0.95  [0.92 – 0.96] | 0.76  [0.67 – 0.84] | 0.35  [0.22 – 0.49] | 0.93  [0.87 – 0.97] |
| PHQ-4 | 0.84  [0.79 – 0.87] | 0.76  [0.68 – 0.83] | 0.75  [0.71 – 0.79] | 0.75  [0.65 – 0.83] | 0.61  [0.46 – 0.75] | 0.74  [0.66 – 0.81] | 0.83  [0.78 – 0.87] | 0.42  [0.34 – 0.51] | 0.95  [0.92 – 0.96] | 0.76  [0.67 – 0.84] | 0.37  [0.24 – 0.51] | 0.93  [0.87 – 0.97] |
| Unmarried | 0.84  [0.79 – 0.87] | 0.74  [0.66 – 0.82] | 0.77  [0.73 – 0.81] | 0.75  [0.65 – 0.83] | 0.61  [0.46 – 0.75] | 0.75  [0.67 – 0.82] | 0.83  [0.79 – 0.87] | 0.44  [0.35 – 0.53] | 0.94  [0.92 – 0.96] | 0.76  [0.67 – 0.84] | 0.37  [0.24 – 0.51] | 0.92  [0.87 – 0.96] |
| Living with children | 0.84  [0.80 – 0.88] | 0.74  [0.65 – 0.81] | 0.77  [0.73 – 0.81] | 0.75  [0.66 – 0.82] | 0.59  [0.44 – 0.73] | 0.77  [0.69 – 0.84] | 0.83  [0.79 – 0.87] | 0.42  [0.33 – 0.50] | 0.95  [0.93 – 0.97] | 0.76  [0.66 – 0.83] | 0.35  [0.22 – 0.49] | 0.94  [0.88 – 0.97] |
| Surgery setting: cardiovascular | 0.83  [0.79 – 0.87] | 0.76  [0.68 – 0.83] | 0.76  [0.71 – 0.80] | 0.75  [0.65 – 0.83] | 0.63  [0.49 – 0.76] | 0.74  [0.66 – 0.81] | 0.83  [0.79 – 0.87] | 0.43  [0.34 – 0.52] | 0.94  [0.92 – 0.96] | 0.76  [0.67 – 0.84] | 0.37  [0.24 – 0.51] | 0.92  [0.87 – 0.96] |
| Living alone | 0.84  [0.80 – 0.88] | 0.76  [0.68 – 0.83] | 0.77  [0.73 – 0.81] | 0.75  [0.65 – 0.82] | 0.61  [0.46 – 0.75] | 0.78  [0.70 – 0.84] | 0.82  [0.78 – 0.86] | 0.41  [0.33 – 0.50] | 0.95  [0.92 – 0.97] | 0.77  [0.68 – 0.84] | 0.35  [0.22 – 0.49] | 0.93  [0.87 – 0.97] |
| Surgery type: heart | 0.83  [0.79 – 0.87] | 0.76  [0.68 – 0.83] | 0.76  [0.72 – 0.80] | 0.75  [0.65 – 0.82] | 0.65  [0.51 – 0.78] | 0.78  [0.71 – 0.85] | 0.84  [0.79 – 0.87] | 0.45  [0.36 – 0.54] | 0.94  [0.92 – 0.96] | 0.76  [0.67 – 0.84] | 0.35  [0.22 – 0.49] | 0.92  [0.87 – 0.96] |
| CHA2DS2-VASc score | 0.84  [0.79 – 0.88] | 0.75  [0.67 – 0.82] | 0.76  [0.71 – 0.80] | 0.75  [0.65 – 0.82] | 0.61  [0.46 – 0.75] | 0.75  [0.67 – 0.82] | 0.83  [0.78 – 0.87] | 0.43  [0.35 – 0.52] | 0.94  [0.92 – 0.96] | 0.76  [0.67 – 0.84] | 0.35  [0.22 – 0.49] | 0.93  [0.87 – 0.97] |
| MoCA | 0.84  [0.79 – 0.87] | 0.75  [0.67 – 0.82] | 0.77  [0.72 – 0.81] | 0.75  [0.65 – 0.83] | 0.59  [0.44 – 0.73] | 0.75  [0.67 – 0.82] | 0.83  [0.79 – 0.87] | 0.42  [0.34 – 0.51] | 0.94  [0.92 – 0.96] | 0.76  [0.67 – 0.84] | 0.35  [0.22 – 0.49] | 0.93  [0.87 – 0.97] |
| PHQ-4 depression | 0.84  [0.79 – 0.88] | 0.75  [0.67 – 0.82] | 0.78  [0.74 – 0.82] | 0.74  [0.65 – 0.82] | 0.61  [0.46 – 0.75] | 0.76  [0.68 – 0.83] | 0.83  [0.78 – 0.86] | 0.42  [0.34 – 0.51] | 0.95  [0.92 – 0.96] | 0.76  [0.67 – 0.84] | 0.35  [0.22 – 0.49] | 0.93  [0.87 – 0.97] |
| EQ-5D-5L | 0.85  [0.80 – 0.88] | 0.76  [0.68 – 0.83] | 0.78  [0.74 – 0.82] | 0.74  [0.65 – 0.82] | 0.63  [0.49 – 0.76] | 0.76  [0.68 – 0.83] | 0.82  [0.77 – 0.86] | 0.46  [0.38 – 0.55] | 0.93  [0.90 – 0.95] | 0.77  [0.68 – 0.84] | 0.30  [0.18 – 0.45] | 0.93  [0.88 – 0.97] |
| PHQ-4 little interest | 0.84  [0.79 – 0.87] | 0.74  [0.66 – 0.82] | 0.76  [0.72 – 0.80] | 0.74  [0.65 – 0.82] | 0.59  [0.44 – 0.73] | 0.75  [0.67 – 0.82] | 0.83  [0.79 – 0.87] | 0.42  [0.34 – 0.51] | 0.95  [0.92 – 0.96] | 0.76  [0.67 – 0.84] | 0.37  [0.24 – 0.51] | 0.92  [0.87 – 0.96] |
| SF-12 pain | 0.84  [0.79 – 0.88] | 0.75  [0.67 – 0.82] | 0.78  [0.73 – 0.82] | 0.74  [0.65 – 0.82] | 0.63  [0.48 – 0.77] | 0.75  [0.67 – 0.82] | 0.83  [0.78 – 0.87] | 0.44  [0.36 – 0.53] | 0.94  [0.92 – 0.96] | 0.76  [0.67 – 0.84] | 0.37  [0.24 – 0.51] | 0.93  [0.87 – 0.97] |
| SF-12 social contacts | 0.84  [0.79 – 0.88] | 0.76  [0.68 – 0.83] | 0.78  [0.74 – 0.82] | 0.74  [0.64 – 0.82] | 0.63  [0.49 – 0.76] | 0.75  [0.67 – 0.82] | 0.83  [0.79 – 0.87] | 0.44  [0.35 – 0.53] | 0.95  [0.92 – 0.97] | 0.75  [0.66 – 0.83] | 0.35  [0.22 – 0.49] | 0.92  [0.87 – 0.96] |
| SF-12 general health | 0.84  [0.80 – 0.88] | 0.79  [0.71 – 0.86] | 0.76  [0.72 – 0.80] | 0.74  [0.64 – 0.82] | 0.63  [0.48 – 0.77] | 0.72  [0.63 – 0.79] | 0.84  [0.80 – 0.88] | 0.42  [0.33 – 0.50] | 0.95  [0.92 – 0.97] | 0.75  [0.66 – 0.83] | 0.37  [0.24 – 0.51] | 0.93  [0.87 – 0.97] |
| General anesthesia using gas | 0.84  [0.79 – 0.87] | 0.77  [0.69 – 0.84] | 0.78  [0.74 – 0.82] | 0.74  [0.64 – 0.82] | 0.65  [0.51 – 0.78] | 0.73  [0.65 – 0.80] | 0.83  [0.78 – 0.86] | 0.42  [0.33 – 0.50] | 0.95  [0.93 – 0.97] | 0.76  [0.66 – 0.83] | 0.33  [0.20 – 0.47] | 0.93  [0.87 – 0.97] |
| EQ-5D-5L pain | 0.84  [0.80 – 0.88] | 0.77  [0.69 – 0.83] | 0.76  [0.71 – 0.80] | 0.74  [0.64 – 0.82] | 0.63  [0.49 – 0.76] | 0.75  [0.67 – 0.82] | 0.82  [0.77 – 0.86] | 0.49  [0.40 – 0.58] | 0.93  [0.90 – 0.95] | 0.76  [0.67 – 0.84] | 0.35  [0.22 – 0.49] | 0.92  [0.86 – 0.96] |
| SF-12 | 0.84  [0.80 – 0.88] | 0.78  [0.69 – 0.84] | 0.78  [0.73 – 0.82] | 0.74  [0.64 – 0.82] | 0.63  [0.49 – 0.76] | 0.75  [0.67 – 0.81] | 0.83  [0.78 – 0.86] | 0.44  [0.36 – 0.53] | 0.95  [0.92 – 0.96] | 0.76  [0.67 – 0.84] | 0.35  [0.22 – 0.49] | 0.92  [0.87 – 0.96] |

Supplementary Table 4: Bias analysis**.** Bias and disparity analysis was performed on the model’s performance on the test set using the aequitas library for python, version 0.42.0. Default settings were used. The male gender and the German language served as the respective reference groups. A significance level of α=0.05 was chosen.

|  | **Gender** | |  |  |
| --- | --- | --- | --- | --- |
|  | **Female (n=93)** | **Male (n=83)** | **Disparity** | **Significance** |
| True Positive Rate | 0.79 | 0.58 | 1.36 | False |
| True Negative Rate | 0.81 | 0.70 | 1.15 | False |
| False Omission Rate | 0.06 | 0.15 | 0.41 | False |
| False Discovery Rate | 0.48 | 0.63 | 0.76 | False |
| False Positive Rate | 0.19 | 0.30 | 0.64 | False |
| False Negative Rate | 0.21 | 0.42 | 0.50 | False |
| Negative Predictive Value | 0.94 | 0.85 | 1.10 | False |
| Precision | 0.52 | 0.37 | 1.41 | False |
| Predicted Positive Ratio*_k_* | 0.49 | 0.51 | 0.97 | False |
| Predicted Positive Ratio*_g_* | 0.31 | 0.36 | 0.86 | False |
|  | **Native Language** | |  |  |
|  | **German (n=160)** | **Other (n=8)** | **Disparity** | **Significance** |
| True Positive Rate | 0.65 | 0.65 | 0.65 | False |
| True Negative Rate | 1.00 | 1.00 | 1.00 | False |
| False Omission Rate | 1.55 | 1.55 | 1.55 | False |
| False Discovery Rate | 0.75 | 0.75 | 0.75 | False |
| False Positive Rate | 0.67 | 0.67 | 0.67 | False |
| False Negative Rate | 0.88 | 0.88 | 0.88 | False |
| Negative Predictive Value | 0.11 | 0.11 | 0.11 | False |
| Precision | 0.00 | 0.00 | 0.00 | False |
| Predicted Positive Ratio*_k_* | 0.00 | 0.00 | 0.00 | False |
| Predicted Positive Ratio*_g_* | 0.58 | 0.58 | 0.58 | False |

Supplementary Table 5: TRIPOD Statement**.** A checklist of items to include in a study developing a clinical prediction tool, listing where specific information can be found in the manuscript.

| **Item** | **Checklist Item** | **Page** |
| --- | --- | --- |
| 1 | Identify the study as developing and/or validating a multivariable prediction model, the target population, and the outcome to be predicted. | 1 |
| 2 | Provide a summary of objectives, study design, setting, participants, sample size, predictors, outcome, statistical analysis, results, and conclusions. | 2 |
| 3a | Explain the medical context (including whether diagnostic or prognostic) and rationale for developing or validating the multivariable prediction model, including references to existing models. | 3 |
| 3b | Specify the objectives, including whether the study describes the development or validation of the model or both. | 3 |
| 4a | Describe the study design or source of data (e.g., randomized trial, cohort, or registry data), separately for the development and validation data sets, if applicable. | 3 |
| 4b | Specify the key study dates, including start of accrual; end of accrual; and, if applicable, end of follow-up. | 3 |
| 5a | Specify key elements of the study setting (e.g., primary care, secondary care, general population) including number and location of centres. | 3 |
| 5b | Describe eligibility criteria for participants. | 3, 4 |
| 5c | Give details of treatments received, if relevant. | Not apllicable |
| 6a | Clearly define the outcome that is predicted by the prediction model, including how and when assessed. | 3, 4 |
| 6b | Report any actions to blind assessment of the outcome to be predicted. | Not applicable |
| 7a | Clearly define all predictors used in developing or validating the multivariable prediction model, including how and when they were measured. | 4, 5, Supplementary Tables 1 and 2 |
| 7b | Report any actions to blind assessment of predictors for the outcome and other predictors. | Not applicable |
| 8 | Explain how the study size was arrived at. | 4 |
| 9 | Describe how missing data were handled (e.g., complete-case analysis, single imputation, multiple imputation) with details of any imputation method | 4 |
| 10a | Describe how predictors were handled in the analyses. | 4, 5 |
| 10b | Specify type of model, all model-building procedures (including any predictor selection), and method for internal validation. | 4, 5 |
| 10d | Specify all measures used to assess model performance and, if relevant, to compare multiple models. | 5 |
| 11 | Provide details on how risk groups were created, if done. | Not applicable |
| 13a | Describe the flow of participants through the study, including the number of participants with and without the outcome and, if applicable, a summary of the follow-up time. A diagram may be helpful. | 6 |
| 13b | Describe the characteristics of the participants (basic demographics, clinical features, available predictors), including the number of participants with missing data for predictors and outcome. | 4, 5 |
| 14a | Specify the number of participants and outcome events in each analysis. | 4, 6 |
| 14b | If done, report the unadjusted association between each candidate predictor and outcome. | Not applicable |
| 15a | Present the full prediction model to allow predictions for individuals (i.e., all regression coefficients, and model intercept or baseline survival at a given time point) | 7, Table 2 |
| 15b | Explain how to the use the prediction model | 6, 7 |
| 16 | Report performance measures (with CIs) for the prediction model | 6 |
| 18 | Discuss any limitations of the study (such as nonrepresentative sample, few events per predictor, missing data) | 8, 9 |
| 19b | Give an overall interpretation of the results, considering objectives, limitations, and results from similar studies, and other relevant evidence | 7, 8, 9 |
| 20 | Discuss the potential clinical use of the model and implications for future research. | 7 |
| 21 | Provide information about the availability of supplementary resources, such as study protocol, Web calculator, and data sets | 9 |
| 22 | Give the source of funding and the role of the funders for the present study | 11 |

Supplementary Table 6: Confusion matrix**.** Confusion matrix for the model when used on the training set (top) and test set (bottom). Precision: Training set 0.49; Test set 0.43. Recall: Training set 0.71; Test set 0.68

| Training Set | | |
| --- | --- | --- |
| **Total**  702 | **Predicted positive**  246 | **Predicted negative**  456 |
| **Positive**  171 | **True positive**  121 | **False negative**  50 |
| **Negative**  531 | **False positive**  125 | **True negative**  406 |
| Test Set | | |
| **Total**  176 | **Predicted positive**  59 | **Predicted negative**  117 |
| **Positive**  38 | **True positive**  26 | **False negative**  12 |
| **Negative**  138 | **False positive**  33 | **True negative**  105 |

## Supplementary Figures


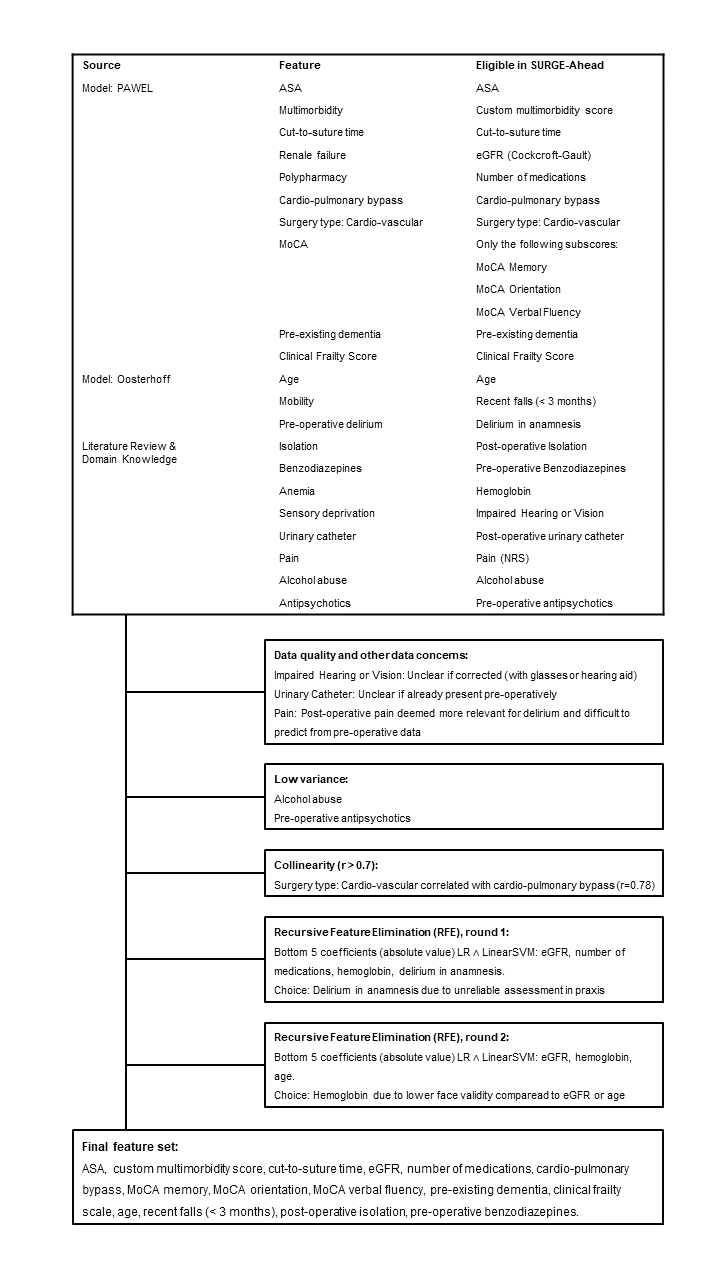


Supplementary Figure 1: Feature Selection Process displayed as a flow chart**.** Candidate Features where identified from previously published models, systematic reviews and domain knowledge. Only Features that are assessed as part of the SURGE-Ahead project were considered eligible for the delirium prediction model. After filtering out features with data quality and other data concerns, low variance or collinearity (r > 0.7), a recursive feature elimination (RFE) was applied: At each step, both a logistic regression model and a linear support vector machine were fitted on the training dataset. For each model, the 5 features with the smallest coefficients were noted and from those present in both models, one was selected for elimination according to our general feature selection strategy (explainability, face validity, possible causal relationship with delirium, ease/reliability of data acquisition), until the final feature set of 15 features remained.


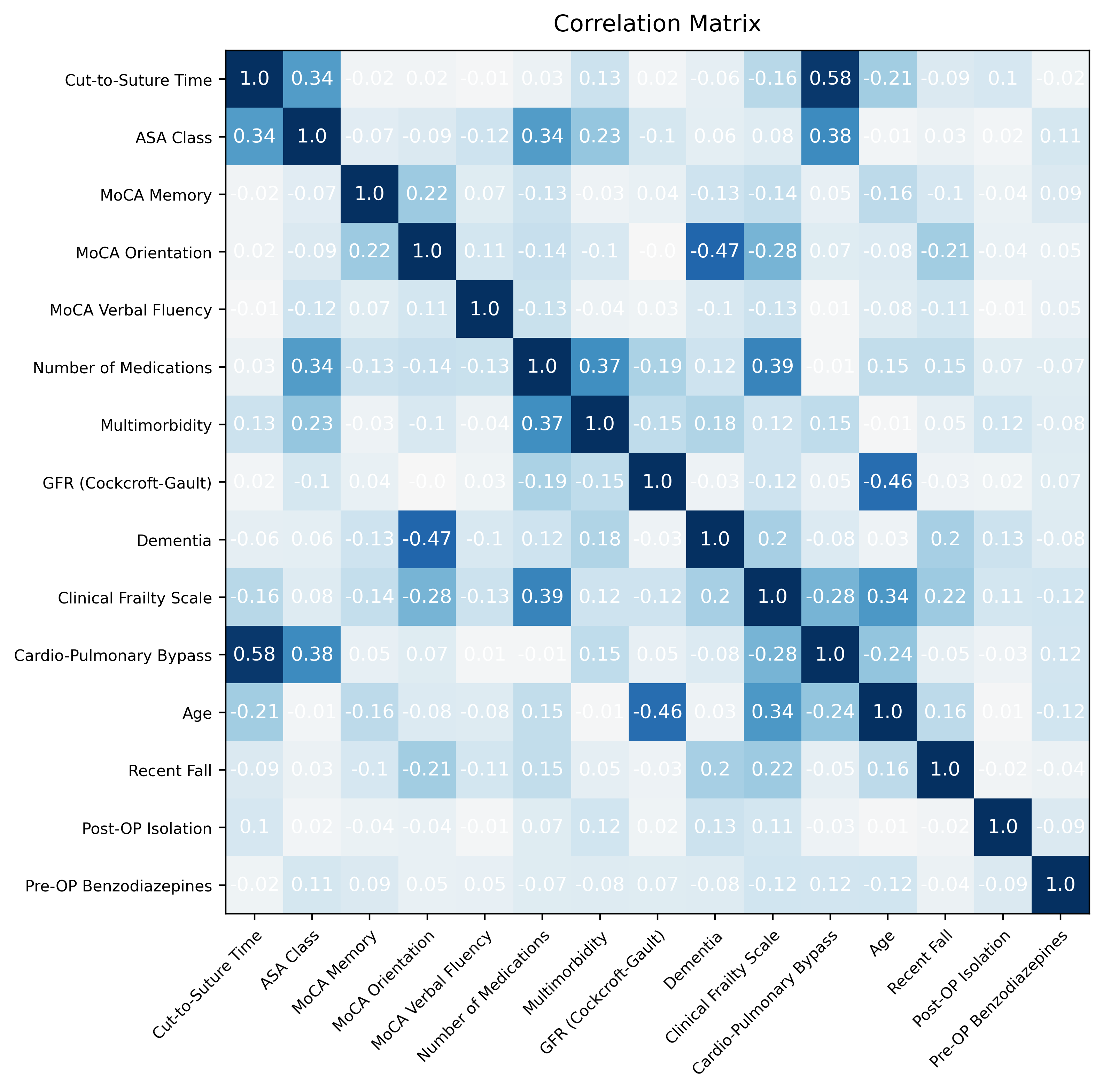


Supplementary Figure 2: Correlation Matrix of the included features**.** The matrix is presented as a heat map, with darker blue colors indicating a higher correlation coefficient. During feature selection, features with a correlation coefficient > 0.7 were discarded.


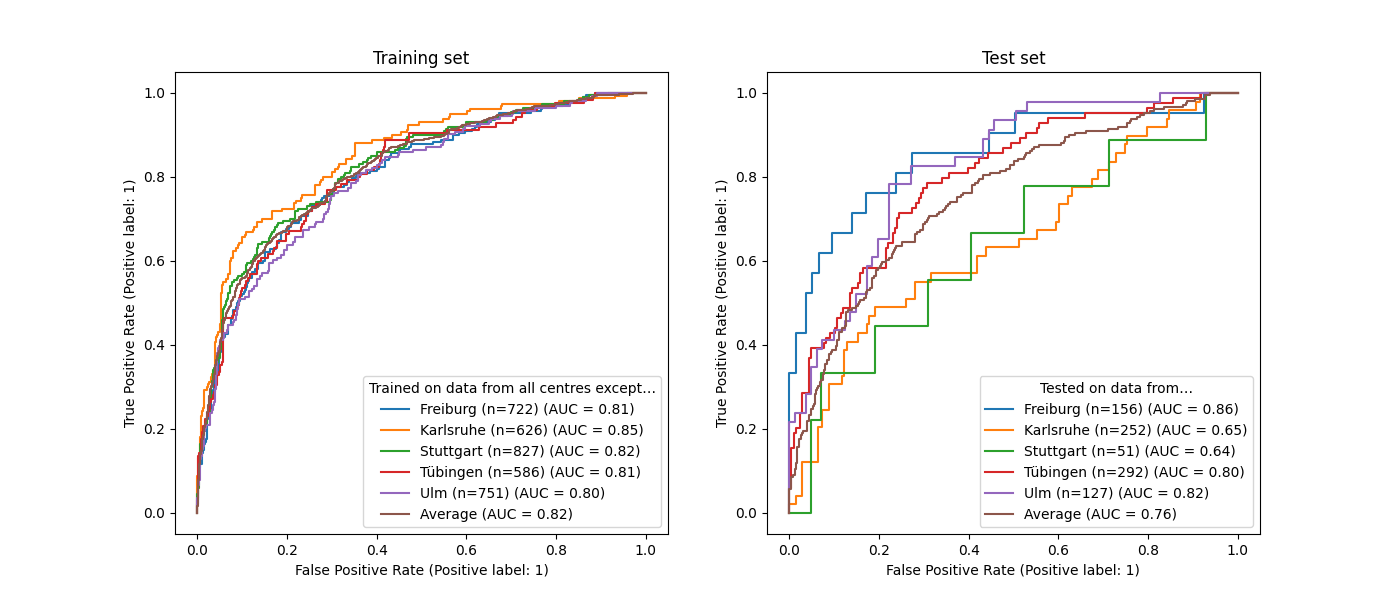
Supplementary Figure 3: Internal cross-validation based on location**.** The model was trained on the complete dataset, leaving out data from a single center, then tested on the data from that held-out center. (AUC: Area under the curve)
