# Supplementary material for: Introducing a machine learning algorithm for delirium prediction—the Supporting SURgery with GEriatric Co-Management and AI project (SURGE-Ahead)
Source: Age Ageing. 2024 May 22;53(5):afae101. doi: 10.1093/ageing/afae101 (PMC11110913; doi:10.1093/ageing/afae101)
Supplement: aa-23-1838-File002_afae101 [file aa-23-1838-file002_afae101.docx]

Introducing a machine learning algorithm for Delirium prediction – the Supporting SURgery with GEriatric Co-Management and AI project (SURGE-Ahead)

# Acknowledgments

The SURGE-Ahead Study Group comprises Anna Ajlani, Karen Andersen-Ranberg, Sophia Andres, Christian Bolenz, Raffael Cintean, Dhayana Dallmeier, Miriam Deniz, Michael Denkinger, Colette Doerr-Harim, Anna Lena Flagmeier, Marina Fotteler, Florian Gebhard, Sandra Graf, Janina Hahn, David Hansen, Daniel Hieber, Thomas Hoffmann, Felix Huettner, Wolfgang Janni, Hans Kestler, Reinhold Kilian, Thomas Kocar, Christoph Leinert, Elena Leinert, Fabia Mangold, Jörg Meerpohl, Christoph Michalski, André Mihaljevic, Annabel Müller-Stierlin, Nadir Nasir, Graziano Onder, Desmond O’Neill, Marcin Orzechowski, Carlos Pankratz, Johannes Schobel, Fabienne Schochter, Konrad Schütze, Tobias Skuban-Eiseler, Vytautas Stasiunaitis, Florian Steger, Walter Swoboda, Adriane Uihlein, Sebastian Voigt-Radloff, Martin Wehling, Pascal Wendel, Felix Wezel, Dennis Wolf, Philip Wolf, Friedemann Zengerling, and Marco Zeptner.

The authors would like to thank all the patients and their relatives who were interviewed and assessed. They also would like to thank especially the data acquisition teams and all staff members at the recruiting centers (see **Supplementary Table 7**) and the PAWEL study steering committee, Prof. G. Eschweiler, Prof. M. Rapp and PD Dr. C. Thomas as well as all researchers of the PAWEL Study group.

Members of the international advisory board who provided input to the study design: Ulf Günther (Department of Anesthesiology, Intensive Care Medicine, and Pain Therapy, University Hospital Oldenburg, Oldenburg, Germany), George A. Kuchel (University of Connecticut Center on Aging, Farmington, CT, United States), Susan Kurrle (Health Care of Older People, Faculty of Medicine, University of Sidney, Sidney, Australia), and Yoanna Skrobik (Department of Medicine, McGill University, Montreal, Quebec, Canada).

PAWEL study group members and collaborators who contributed to this work: PAWEL Study Group Steering and Executive Committee: Gerhard Eschweiler (Head), Michael Rapp, Christine Thomas (Steering Committee) and Christine A.F. von Arnim, Michael Denkinger, (Ulm University), Brigitte R. Metz, Lars O. Conzelmann (ViDiA and Helios Karlsruhe) Felix Kentischer, Christoph Maurer (Freiburg University), Stefanie Joos, Cindy Boden (Tübingen University) Sören Wagner, Eva Mennig (Klinikum Stuttgart).

PAWEL Study Group Data Management and Statistical Analysis Core: Michael Rapp (Head), Andreas Häusler, Friederike Deeken, Bernd Förstner, Alba Sanchez and Susanne Schulze (all: Department of Social and Preventive Medicine, University of Potsdam, Potsdam, Germany.

PAWEL Study Group Economic Analysis: Jürgen Wasem (Head), and Anja Neumann (Institute for Healthcare Management and Research); (all University Duisburg-Essen, Essen, Germany); AOK Baden-Württemberg, Stuttgart, Germany: Julia Frankenhauser-Mannuß and Julia Pick.

PAWEL Study Group Intervention Development, Training and Supervision Core: PD Dr. Christine Thomas (Head); Stefan Blumenrode (Psychiatric Nurses' Qualification Institute); Carola Bruns, Juliane Spank, Sarah Weller, and Eva Mennig (Department of Old Age Psychiatry and Psychotherapy) all Klinikum Stuttgart, Germany. Christine von Arnim, University Hospital Tübingen: Florian Metzger (Department of Psychiatry and Psychotherapy); Andreas Straub (Department of Anesthesiology); Tobias Krüger (Department of Heart Surgery); Felix Bausenhart (Department of Orthopedics); and Petra Renz (Nursing Department). Klinikum Stuttgart, Stuttgart, Germany: Andreas Walther, Sören Wagner (Department of Anaesthesiology and Intensive Care, Katharinenhospital); Carola Bruns, Juliane Spank, and Eva Mennig (Department of Old Age Psychiatry and Psychotherapy). University of Freiburg, Freiburg, Germany: Markus Martin (Department of Neurology and Neurophysiology); Bernhard Heimbach, and Sebastian Voigt-Radloff (Center for Geriatric Medicine and Gerontology (ZGGF). University Hospital Ulm, of Ulm, Germany: Heiko Reichel (Department of Orthopedics); Andreas Liebold (Department of Cardiothoracic and Vascular Surgery); and Simone Brefka (Agaplesion Bethesda Clinic, Geriatric Medicine, Ulm University and Geriatric Center Ulm). Study center in Karlsruhe, Germany: Stephan Kirschner (Department of Orthopeadics, ViDia Christian Clinics Karlsruhe); Nina Stober (Geriatric Center Karlsruhe, ViDia Christian Clinics Karlsruhe); and Uwe Mehlhorn (Helios Clinic for Cardiac Surgery).

Study data were collected and managed by using SecuTrial® electronic data capture tools hosted at the University of Potsdam. The PAWEL study is funded from 2017 to 2020 by the Innovationsfonds from the Gemeinsamer Bundesausschuss (G-BA) Kennzeichen O1VSF-16016

# Ethics statement

The studies involving human participants were reviewed and approved by the Ethics Commission of the Faculty of Medicine of the Eberhard-Karls University and University Hospital Tübingen with number 233/2017BO1 on October 12, 2017 and by the Ethics Commission of the University of Potsdam with number 38/2017 on December 11, 2017. The patients/participants provided their written informed consent to participate in this study.

# Data availability statement

The python code is available on GitHub (https://github.com/IfGF-UUlm/SURGE-Ahead_Delirium), the data is is property of the PAWEL project and will be released publicly at their discretion [2].
